# The Coming of Age of Nucleic Acid Vaccines during COVID-19

**DOI:** 10.1128/msystems.00928-22

**Published:** 2023-03-02

**Authors:** Halie M. Rando, Ronan Lordan, Likhitha Kolla, Elizabeth Sell, Alexandra J. Lee, Nils Wellhausen, Amruta Naik, Jeremy P. Kamil, COVID-19 Review Consortium, Anthony Gitter, Casey S. Greene

**Affiliations:** a Department of Systems Pharmacology and Translational Therapeutics, University of Pennsylvania, Philadelphia, Pennsylvania, USA; b Department of Biochemistry and Molecular Genetics, University of Colorado Anschutz School of Medicine, Aurora, Colorado, USA; c Center for Health AI, University of Colorado Anschutz School of Medicine, Aurora, Colorado, USA; d Department of Biomedical Informatics, University of Colorado Anschutz School of Medicine, Aurora, Colorado, USA; e Institute for Translational Medicine and Therapeutics, Perelman School of Medicine, University of Pennsylvania, Philadelphia, Pennsylvania, USA; f Department of Medicine, Perelman School of Medicine, University of Pennsylvania, Philadelphia, Pennsylvania, USA; g Perelman School of Medicine, University of Pennsylvania, Philadelphia, Pennsylvania, USA; h Children’s Hospital of Philadelphia, Philadelphia, Pennsylvania, USA; i Department of Microbiology and Immunology, Louisiana State University Health Sciences Center Shreveport, Shreveport, Louisiana, USA; j Department of Biostatistics and Medical Informatics, University of Wisconsin—Madison, Madison, Wisconsin, USA; k Morgridge Institute for Research, Madison, Wisconsin, USA; l Childhood Cancer Data Lab, Alex’s Lemonade Stand Foundation, Philadelphia, Pennsylvania, USA; University of California San Diego

**Keywords:** COVID-19, DNA vaccines, SARS-CoV-2, mRNA vaccines, nucleic acid vaccines, review, vaccine equity, vaccines

## Abstract

In the 21st century, several emergent viruses have posed a global threat. Each pathogen has emphasized the value of rapid and scalable vaccine development programs. The ongoing severe acute respiratory syndrome coronavirus 2 (SARS-CoV-2) pandemic has made the importance of such efforts especially clear. New biotechnological advances in vaccinology allow for recent advances that provide only the nucleic acid building blocks of an antigen, eliminating many safety concerns. During the COVID-19 pandemic, these DNA and RNA vaccines have facilitated the development and deployment of vaccines at an unprecedented pace. This success was attributable at least in part to broader shifts in scientific research relative to prior epidemics: the genome of SARS-CoV-2 was available as early as January 2020, facilitating global efforts in the development of DNA and RNA vaccines within 2 weeks of the international community becoming aware of the new viral threat. Additionally, these technologies that were previously only theoretical are not only safe but also highly efficacious. Although historically a slow process, the rapid development of vaccines during the COVID-19 crisis reveals a major shift in vaccine technologies. Here, we provide historical context for the emergence of these paradigm-shifting vaccines. We describe several DNA and RNA vaccines in terms of their efficacy, safety, and approval status. We also discuss patterns in worldwide distribution. The advances made since early 2020 provide an exceptional illustration of how rapidly vaccine development technology has advanced in the last 2 decades in particular and suggest a new era in vaccines against emerging pathogens.

**IMPORTANCE** The SARS-CoV-2 pandemic has caused untold damage globally, presenting unusual demands on but also unique opportunities for vaccine development. The development, production, and distribution of vaccines are imperative to saving lives, preventing severe illness, and reducing the economic and social burdens caused by the COVID-19 pandemic. Although vaccine technologies that provide the DNA or RNA sequence of an antigen had never previously been approved for use in humans, they have played a major role in the management of SARS-CoV-2. In this review, we discuss the history of these vaccines and how they have been applied to SARS-CoV-2. Additionally, given that the evolution of new SARS-CoV-2 variants continues to present a significant challenge in 2022, these vaccines remain an important and evolving tool in the biomedical response to the pandemic.

## INTRODUCTION

Severe acute respiratory syndrome coronavirus 2 (SARS-CoV-2) emerged at the end of 2019 and soon spread around the world. In response, the Coalition for Epidemic Preparedness Innovations quickly began coordinating global health agencies and pharmaceutical companies to develop vaccines, as vaccination is one of the primary approaches available to combat the effects of a virus. Vaccines can bolster the immune response to a virus at both the individual and population levels, thereby reducing fatalities and severe illness and potentially driving a lower rate of infection even for a highly infectious virus like SARS-CoV-2. However, vaccines have historically required a lengthy development process due to both experimental and regulatory demands.

As we review in a companion article (1), vaccine technologies prior to the COVID-19 pandemic were largely based on triggering an immune response by introducing a virus or one of its components. Such vaccines are designed to induce an adaptive immune response without causing the associated viral illness. Each time a virus emerges that poses a significant global threat, as has happened several times over the past 20 years, the value of a rapid vaccine response is underscored. With progressive biotechnological developments, this objective has become increasingly tangible.

In the current century, significant advances in vaccine development have largely been built on genomics, as is somewhat unsurprising given the impact of the genomic revolution across all biology. This shift toward nucleic acid-based technologies opens a new frontier in vaccinology, where just the sequence encoding an antigen can be introduced to induce an immune response. While other platforms can carry some risks related to introducing all or part of a virus (1), nucleic acid-based platforms eliminate these risks entirely. Additionally, vaccine technologies that could be adjusted for novel viral threats are appealing because this modular approach would mean they could enter trials quickly in response to a new pathogen of concern.

## HONING A 21ST-CENTURY RESPONSE TO EMERGENT VIRAL THREATS

Recently, vaccine technologies have been developed and refined in response to several epidemics that did not reach the level of destruction caused by COVID-19. Emergent viral threats of the 21st century include severe acute respiratory syndrome (SARS), the H1N1 influenza strain known as swine flu, Middle East respiratory syndrome (MERS), Ebola virus disease, COVID-19, and, most recently, monkeypox, all of which have underscored the importance of a rapid global response to a new infectious virus. Because the vaccine development process has historically been slow, the use of vaccines to control most of these epidemics was limited.

One of the more successful recent vaccine development programs was for H1N1 influenza. This program benefited from the strong existing infrastructure for influenza vaccines along with the fact that regulatory agencies had determined that vaccines produced using egg- and cell-based platforms could be licensed under the regulations used for a strain change (2). Although a monovalent H1N1 vaccine was not available before the pandemic peaked in the United States and Europe, it became available soon afterward as a stand-alone vaccine that was eventually incorporated into commercially available seasonal influenza vaccines (2). Critiques of the production and distribution of the H1N1 vaccine have stressed the need for alternative development and manufacturing platforms that can be readily adapted to new pathogens.

Efforts to develop such approaches had been undertaken prior to the COVID-19 pandemic. DNA vaccine development efforts began for SARS-CoV-1 but did not proceed past animal testing (3). Likewise, the development of virus-vectored Ebola virus vaccines was undertaken, but the pace of vaccine development was behind the spread of the virus from early on (4). Although a candidate Ebola vaccine, V920, showed promise in preclinical and clinical testing, it did not receive breakthrough therapy designation until the summer of 2016, by which time the Ebola outbreak was winding down (5). Therefore, the COVID-19 pandemic has been the first case where vaccines have been available early enough to significantly influence outcomes at the global scale.

The pandemic caused by SARS-CoV-2 has highlighted a confluence of circumstances that positioned vaccine development as a key player in efforts to control the virus and mitigate its damage. This virus did not follow the same trajectory as other emergent viruses of recent note, such as SARS-CoV-1, MERS-CoV, and Ebola virus, none of which presented a global threat for such a sustained duration (see visualization in reference 6). The spread of SARS-CoV-2 has remained out of control in many parts of the world into 2022, especially with the emergence of novel variants exhibiting increased rates of transmission (7). While, for a variety of reasons, SARS-CoV-2 was not controlled as rapidly as the viruses underlying prior 21st-century epidemics, vaccine development technology had also progressed based on these and other prior viral threats to the point that a rapid international vaccine development response was possible.

## DEVELOPMENT OF COVID-19 VACCINES USING DNA AND RNA PLATFORMS

**FIG 1:**
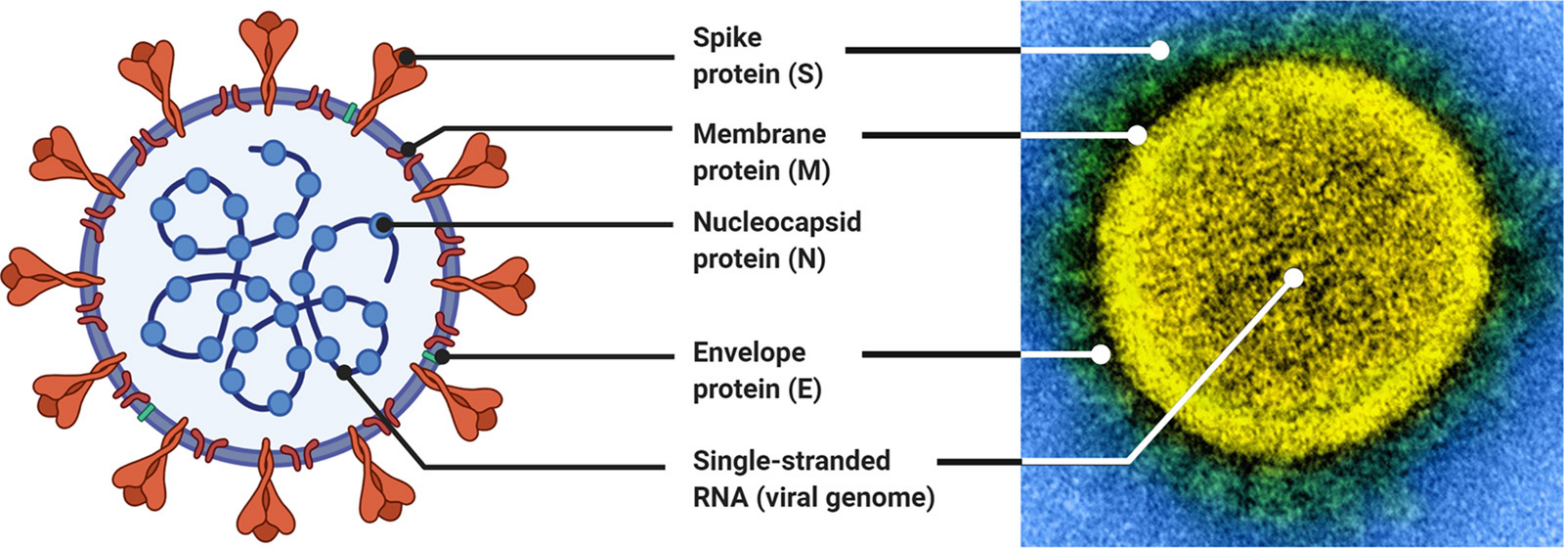
Structure of SARS-CoV-2. The development of vaccines depends on the immune system recognizing the virus. Here, the structure of SARS-CoV-2 is represented both in the abstract and against a visualization of the virion. The abstracted visualization was made using BioRender (https://biorender.com) using the template “Human Coronavirus Structure” by BioRender (August 2020) (234). The microscopy was conducted by the National Institute of Allergy and Infectious Diseases (235).

Vaccine development programs for COVID-19 emerged very quickly. The first administration of a dose of a COVID-19 vaccine to a human trial participant occurred on 16 March 2020 (8, 9), marking an extremely rapid response to the emergence of SARS-CoV-2. Within a few weeks of this first trial launching, at least 78 vaccine development programs were active (9), and by September 2020, there were over 180 vaccine candidates against SARS-CoV-2 in development (10). As of 7 October 2022, 47 SARS-CoV-2 vaccines have been approved worldwide and 27 are being administered throughout the world, with 13.0 billion doses administered across 223 countries. The first critical step toward developing a vaccine against SARS-CoV-2 was characterizing the viral target, which happened extremely early in the COVID-19 outbreak with the sequencing and dissemination of the viral genome in early January 2020 (11) (Fig. 1). This genomic information allowed for an early identification of the sequence of the spike (S) protein (Fig. 1), which is the antigen and induces an immune response (12, 13).

During the development process, one measure used to assess whether a vaccine candidate is likely to provide protection is serum neutralizing activity (14). This assay evaluates the presence of antibodies that can neutralize, or prevent infection by, the virus in question. Often, titration is used to determine the extent of neutralization activity. However, unlike in efforts to develop vaccines for prior viral threats, the duration of the COVID-19 pandemic has made it possible to also test vaccines in phase III trials in which the effect of the vaccines on a cohort’s likelihood of contracting SARS-CoV-2 was evaluated.

## THEORY AND IMPLEMENTATION OF NUCLEIC ACID VACCINES

Biomedical research in the 21st century has been significantly influenced by the genomic revolution. While traditional methods of vaccine development, such as inactivated whole viruses, are still used today (1), vaccine development is no exception. The shift toward omics-based approaches to vaccine development began to take hold with the meningococcal type B vaccine, which was developed using reverse vaccinology in the early 2010s (15, 16). Under this approach, the genome of a pathogen is screened to identify potential vaccine targets (16), and pathogens of interest are then expressed *in vitro* and tested in animal models to determine their immunogenicity (16). In this way, the genomic revolution catalyzed a fundamental shift in the development of vaccines. Such technologies could revolutionize the role of vaccines given their potential to address one of the major limitations of vaccines today and facilitate the design of therapeutic, rather than just prophylactic, vaccines (17).

Nucleic acid-based approaches share an underlying principle: a vector that delivers the information needed to produce an antigen. When the host cells manufacture the antigen, it can then trigger an immune response. The fact that no part of the virus is introduced aside from the genetic code of the antigen means that these vaccines carry no risk of infection. Such approaches build on subunit vaccination strategies, in which a component of a virus (e.g., an antigenic protein) is delivered by the vaccine. Platforms based on genomic sequencing began to be explored beginning in the 1980s as genetic research became increasingly feasible. Advances in genetic engineering allowed for gene sequences of specific viral antigens to be grown *in vitro* (18). Studies also demonstrated that model organisms could be induced to construct antigens that would trigger an immune response (19–21). These two developments sparked interest in whether it could be possible to identify any or all of the antigens encoded by a virus’s genome and train the immune response to recognize them.

The delivery and presentation of antigens are fundamental to inducing immunity against a virus. Vaccines that deliver nucleic acids allow the introduction of foreign substances to the body to induce both humoral and cellular immune responses (22). Delivering a nucleic acid sequence to host cells allows the host to synthesize an antigen without exposure to a viral threat (22). Host-synthesized antigens can activate both humoral and cellular immunity (22), as they can be presented in complex with major histocompatibility complex (MHC) I and II, which can activate either T or B cells (22). In contrast, prior approaches activated only MHC II (21). Because these vaccines encode specific proteins, providing many of the benefits of a protein subunit vaccine, they do not carry any risk of DNA being live, replicating, or spreading, and their manufacturing process lends itself to scalability (22). Here, opportunities can be framed in terms of the central dogma of genetics: instead of directly providing the proteins from the infectious agents, vaccine developers are exploring the potential for the delivery of DNA or RNA to induce the cell to produce proteins from the virus that in turn induce a host immune response.

## CROSS-PLATFORM CONSIDERATIONS IN VACCINE DEVELOPMENT

Certain design decisions are relevant to vaccine development across multiple platforms. One applies to the platforms that deliver the antigen, which in the case of SARS-CoV-2 vaccines is the S protein. The prefusion conformation of the S protein, which is the structure before the virus fuses to the host cell membrane, is metastable (23), and the release of energy during membrane fusion drives this process forward following destabilization (24, 25). Due to the significant conformational changes that occur during membrane fusion (26–28), S protein immunogens that are stabilized in the prefusion conformation are of particular interest, especially because a prefusion stabilized MERS-CoV S antigen was found to elicit an improved antibody response (29). Moreover, the prefusion conformation offers an opportunity to target S2, a region of the S protein that accumulates mutations at a lower rate (29–31) (as also reviewed in [7]). Vaccine developers can stabilize the prefusion conformer by selecting versions of the S protein containing mutations that lock the position (32). The immune response to the spike protein when it is stabilized in this conformation is improved over other S structures (33). Thus, vaccines that use this prefusion stabilized conformation are expected to not only offer improved immunogenicity but also be more resilient to the accumulation of mutations in SARS-CoV-2.

Another cross-platform consideration is the use of adjuvants. Adjuvants include a variety of molecules or larger microbe-related products that affect the immune system broadly or an immune response of interest. They can either be comprised of or contain immunostimulants or immunomodulators. Adjuvants are sometimes included within vaccines in order to enhance the immune response. Different adjuvants can regulate different types of immune responses, so the type or combination of adjuvants used in a vaccine will depend on both the type of vaccine and concern related to efficacy and safety. A variety of possible mechanisms for adjuvants have been investigated (34–36).

Due to viral evolution, vaccine developers are in an arms race with a pathogen that benefits from mutations that reduce its susceptibility to adaptive immunity. The evolution of several variants of concern (VOC) presents significant challenges for vaccines developed based on the index strain identified in Wuhan in late 2019. We discuss these variants in depth elsewhere in the COVID-19 Review Consortium project (37). To date, the most significant variants of concern identified are Alpha (2020), Beta (2020), Gamma (2020), Delta (2021), Omicron (2021), and related Omicron subvariants (2022). The effectiveness or efficacy (i.e., trial or real-world prevention, respectively) of vaccines in the context of these variants is discussed where information is available.

## DNA VACCINE PLATFORMS

DNA vaccine technologies have developed slowly over the past 30 years. These vaccines introduce a vector containing a DNA sequence that encodes an antigen(s) selected to induce a specific immune response (21). Early attempts revealed issues with low immunogenicity (19, 21, 38). Additionally, initial skepticism about the approach suggested that DNA vaccines might bind to the host genome or induce autoimmune disease (22, 39), but preclinical and clinical studies have consistently disproved this hypothesis and indicated DNA vaccines to be safe (38). Another concern, antibiotic resistance introduced during the plasmid selection process, did remain a concern during this initial phase of development (22), but this issue was resolved through strategic vector design (40, 41). However, for many years, the immunogenicity of DNA vaccines failed to reach expectations (22). Several developments during the 2010s led to greater efficacy of DNA vaccines (22). However, no DNA vaccines had been approved for use in humans prior to the COVID-19 pandemic (38, 42). As of 7 October 2022, 10 vaccines have been approved worldwide (Table 1). These vaccines fall into two categories, vaccines that are vectored with a plasmid and those that are vectored with another virus.

**TABLE 1:** DNA vaccines approved in at least one country (237) as of 7 October 2022

Vaccine	Company	Platform
iNCOVACC	Bharat Biotech	Nonreplicating viral vector
Ad5-nCoV-IH	CanSino	Nonreplicating viral vector
Convidecia	CanSino	Nonreplicating viral vector
Gam-COVID-Vac	Gamaleya	Nonreplicating viral vector
Sputnik Light	Gamaleya	Nonreplicating viral vector
Sputnik V	Gamaleya	Nonreplicating viral vector
Jcovden	Janssen (Johnson & Johnson)	Nonreplicating viral vector
Vaxzevria	Oxford/AstraZeneca	Nonreplicating viral vector
Covishield (Oxford/ AstraZeneca formulation)	Serum Institute of India	Nonreplicating viral vector
ZyCoV-D	Zydus Cadila	Plasmid vector

### Plasmid-vectored DNA vaccines.

Many DNA vaccines use a plasmid vector-based approach, where the sequence encoding the antigen(s) against which an immune response is sought is cultivated in a plasmid and delivered directly to an appropriate tissue (43). Plasmids can also be designed to act as adjuvants by targeting essential regulators of pathways such as the inflammasome or simply just specific cytokines (39, 44). The DNA itself may also stimulate the innate immune response (21, 41). Once the plasmid brings the DNA sequence to an antigen-presenting cell (APC), the host machinery can be used to construct antigen(s) from the transported genetic material, and the body can then synthesize antibodies in response (22). The vectors are edited to remove extra sequences (41). These types of manufacturing advances have improved the safety and throughput of this platform (41).

**(i) Prior applications.** In the 1990s and 2000s, DNA vaccines delivered via plasmids sparked significant scientific interest, leading to a large number of preclinical trials (22). Early preclinical trials primarily focused on long-standing disease threats, including viral diseases such as rabies and parasitic diseases such as malaria, and promising results led to phase I testing of the application of this technology to human immunodeficiency virus (HIV), influenza, malaria, and other diseases of concern during this period (22). Although they were well tolerated, these early attempts to develop vaccines were generally not very successful in inducing immunity to the target pathogen, with either limited T cell or limited neutralizing antibody responses observed (22).

Early plasmid-vectored DNA vaccine trials targeted HIV and subsequently diseases of worldwide importance such as malaria and hepatitis B (45). The concern with these early development projects was immunogenicity, not safety (45). Around the turn of the millennium, a hepatitis B vaccine development program demonstrated that these vaccines can induce both antibody and cellular immune responses (46). Prior to COVID-19, however, plasmid-vectored DNA vaccines had been approved for commercial use only in veterinary populations (47–49). Between 2005 and 2006, several DNA vaccines were developed for nonhuman animal populations, including against viruses such as a rhabdovirus in fish (50), porcine reproductive and respiratory syndrome virus (51), and West Nile virus in horses (52). Within the past 5 years, additional plasmid-vectored vaccines for immunization against viruses were developed against a herpesvirus (in mice) (53) and an alphavirus (in fish) (54).

**(ii) Applications to COVID-19.** Several plasmid-vectored DNA vaccines have been developed against COVID-19 (Table 1). In fact, the ZyCoV-D vaccine developed by India’s Zydus Cadila is the first plasmid-vectored DNA vaccine to receive approval or to be used in human medicine (55–57). Another plasmid-vectored DNA vaccine, INO-4800 (58), was developed by Inovio Pharmaceuticals Technology and uses electroporation as an adjuvant. Electroporation was developed as a solution to the issue of limited immunogenicity by increasing the permeability of cell membranes by delivering electrical pulses (59). It has been shown that electroporation can enhance vaccine efficacy (VE) up to 100-fold, as measured by increases in antigen-specific antibody titers (60). The temporary formation of pores through electroporation facilitates the successful transportation of macromolecules into cells, allowing cells to robustly take up INO-4800 for the production of an antibody response. For INO-4800, a plasmid-vectored vaccine is delivered through intradermal injection, which is then followed by electroporation with a device known as CELLECTRA (61). The safety of the CELLECTRA device has been studied for over 7 years, and these studies support the further development of electroporation as a safe vaccine delivery method (59).

These vaccines therefore represent implementations of a new platform technology. In particular, they offer the advantage of a temperature-stable vaccine, facilitating worldwide administration (62). Although an exciting development in DNA vaccines, the cost of vaccine manufacturing and electroporation may make scaling the use of this technology for prophylactic use for the general public difficult.

**(iii) Trial safety and immunogenicity.** The INO-4800 trials began with a phase I trial evaluating two different doses administered as a two-dose series (61). This trial found the vaccine to be safe, with only six adverse events (AEs) reported by 39 participants, all grade 1, and effective, with all but 3 participants of 38 developing serum IgG binding titers to the SARS-CoV-2 S protein (61). A phase II trial of 401 adults at high risk of exposure to SARS-CoV-2 similarly supported the safety and efficacy of INO-4800. Only one treatment-related AE was observed, and the vaccine was found to be associated with a significant increase in neutralizing activity (62). Results of phase III trials are not yet available (63–66).

Trials of ZyCoV-D have progressed further. This vaccine uses a plasmid to deliver the expression-competent spike protein and IgE signal peptides to the vaccinee (67). During the phase I trial, vaccination with a needle versus a needle-free injection system was evaluated, and the vaccine can now be administered without a needle (55, 56). A phase III trial enrolling over 27,000 patients found no difference in AEs between the placebo and treatment groups and estimated the efficacy of ZyCoV-D to be 66.6% (68). It was authorized for people ages 12 and older (57). The highly portable design offers advantages over traditional vaccines (67), especially as the emergence of variants continues to challenge the effectiveness of vaccines. As of August 2022, ZyCoV-D has only been approved in India (69) and is not tracked by Our World in Data (70).

**(iv) Real-world safety and effectiveness.** In terms of the ability of plasmid-vectored vaccines to neutralize VOC, varying information is available. The situation for ZyCoV-D is somewhat different, as the phase III trial occurred during the Delta wave in India (68). At present, no major press releases have addressed the vaccine’s ability to neutralize Omicron and related VOC, but reporting suggests that the manufacturers were optimistic about the vaccine in light of the Omicron variant as of late 2021 (71).

As for INO-4800, studies have examined whether the induced immune response can neutralize existing VOC. They assessed neutralization of several VOC relative to the index strain and found no difference in neutralization between the index strain and the Gamma VOC (P.1) (72). However, neutralization of the Alpha and Beta VOC was significantly lower (approximately two and seven times, respectively) (72). These findings are in line with the shifts in effectiveness reported for other vaccines (1). In addition to loss of neutralizing activity due to viral evolution, studies have also evaluated the decline in neutralizing antibodies (nAbs) induced by INO-4800 over time. Levels of nAbs remained statistically significant relative to the prevaccination baseline for 6 months (73). Administration of a booster dose induced a significant increase of titers relative to their prebooster levels (73). Given the timing of this trial (enrollment between April and July 2020), it is unlikely that participants were exposed to VOC associated with decreased efficacy.

In light of the emergence of VOC against which many vaccines show lower effectiveness, Inovio Pharmaceuticals began to develop a new vaccine with the goal of improving robustness against known and future VOC (74). Known as INO-4802, this vaccine was designed to express a pan-spike immunogen (75). Booster studies in rodents (76) and nonhuman primates (75) suggest that it may be more effective than INO-4800 in providing immunity to VOC such as Delta and Omicron when administered as part of a heterologous boost regimen, although boosting with INO-4800 was also very effective in increasing immunity in rhesus macaques (75). Therefore, boosting is likely to be an important strategy for this vaccine, especially as the virus continues to evolve.

### Virus-vectored DNA vaccines.

Plasmids are not the only vector that can be used to deliver sequences associated with viral antigens. Genetic material from the target virus can also be delivered using a second virus as a vector. Viral vectors have emerged as a safe and efficient method to furnish the nucleotide sequences of an antigen to the immune system (77). The genetic content of the vector virus is often altered to prevent it from replicating, but replication-competent viruses can also be used under certain circumstances (78). Once the plasmid or viral vector brings the DNA sequence to an APC, the host machinery can be used to construct antigen(s) from the transported genetic material, and the host can then synthesize antibodies in response (22).

One of the early viral vectors explored was adenovirus, with serotype 5 (Ad5) being particularly effective (22). This technology rose in popularity during the 2000s due to its being more immunogenic in humans and nonhuman primates than plasmid-vectored DNA vaccines (22). In the 2000s, interest also arose in utilizing simian adenoviruses as vectors because of the reduced risk that human vaccine recipients would have prior exposure resulting in adaptive immunity (22, 79), and chimpanzee adenoviruses were explored as potential vectors in the development of a vaccine against MERS-CoV (80).

Today, various viral-vector platforms including poxviruses (81, 82), adenoviruses (83), and vesicular stomatitis viruses (84, 85) are being developed. Viral vector vaccines are able to induce both an antibody and cellular response; however, the response is limited due to the immunogenicity of the viral vector used (83, 86). An important consideration in identifying potential vectors is the immune response to the vector. Both the innate and adaptive immune responses can potentially respond to the vector, limiting the ability of the vaccine to transfer information to the immune system (87). Different vectors are associated with different levels of reactogenicity; for example, adenoviruses elicit a much stronger innate immune response than replication-deficient adeno-associated viruses derived from parvoviruses (87). Additionally, using a virus circulating widely in human populations as a vector presents additional challenges because vaccine recipients may already have developed an immune response to the vector (88). Furthermore, repeated exposure to adenoviruses via virus-vectored DNA vaccines may increase reactivity to these vectors over time, presenting a challenge that will need to be considered in long-term development of these vaccines (89, 90).

**(i) Prior applications.** There are several viral vector vaccines that are available for veterinary use (22, 91), but prior to the COVID-19 pandemic, only one viral vector vaccine was approved by the U.S. Food and Drug Administration (FDA) for use in humans. This vaccine is vectored with a recombinant vesicular stomatitis virus and targeted against the Ebola virus (92). Additionally, several phase I and phase II clinical trials for other vaccines are ongoing (77), and the technology is currently being explored for its potential against numerous infectious diseases, including malaria (93, 94), Ebola (95–97), and HIV (98, 99).

The threat of MERS and SARS initiated interest in the application of viral vector vaccines to human coronaviruses (80), but efforts to apply this technology to these pathogens had not yet led to a successful vaccine candidate. In the mid-2000s to late 2000s, adenovirus-vectored vaccines against SARS were found to induce SARS-CoV-specific IgA in the lungs of mice (100) but were later found to offer incomplete protection in ferret models (101). The Gamaleya National Center of Epidemiology and Microbiology in Moscow sought to use an adenovirus platform for the development of vaccines for MERS-CoV and Ebola virus, although neither of the previous vaccines was internationally licensed (102).

In 2017, results were published from an initial investigation of two vaccine candidates against MERS-CoV containing the MERS-CoV *S* gene vectored with chimpanzee adenovirus, Oxford University #1 (ChAdOx1), a replication-deficient chimpanzee adenovirus (103). This study reported that a candidate containing the complete S protein sequence induced a stronger neutralizing antibody response in mice than candidates vectored with modified vaccinia virus Ankara.

The candidate was pursued in additional research, and in the summer of 2020, results of two studies were published. The first reported that a single dose of ChAdOx1 MERS induced an immune response and inhibited viral replication in macaques (104). The second reported promising results from a phase I trial that administered the vaccine to adults and measured safety, tolerability, and immune response (105).

**FIG 2:**
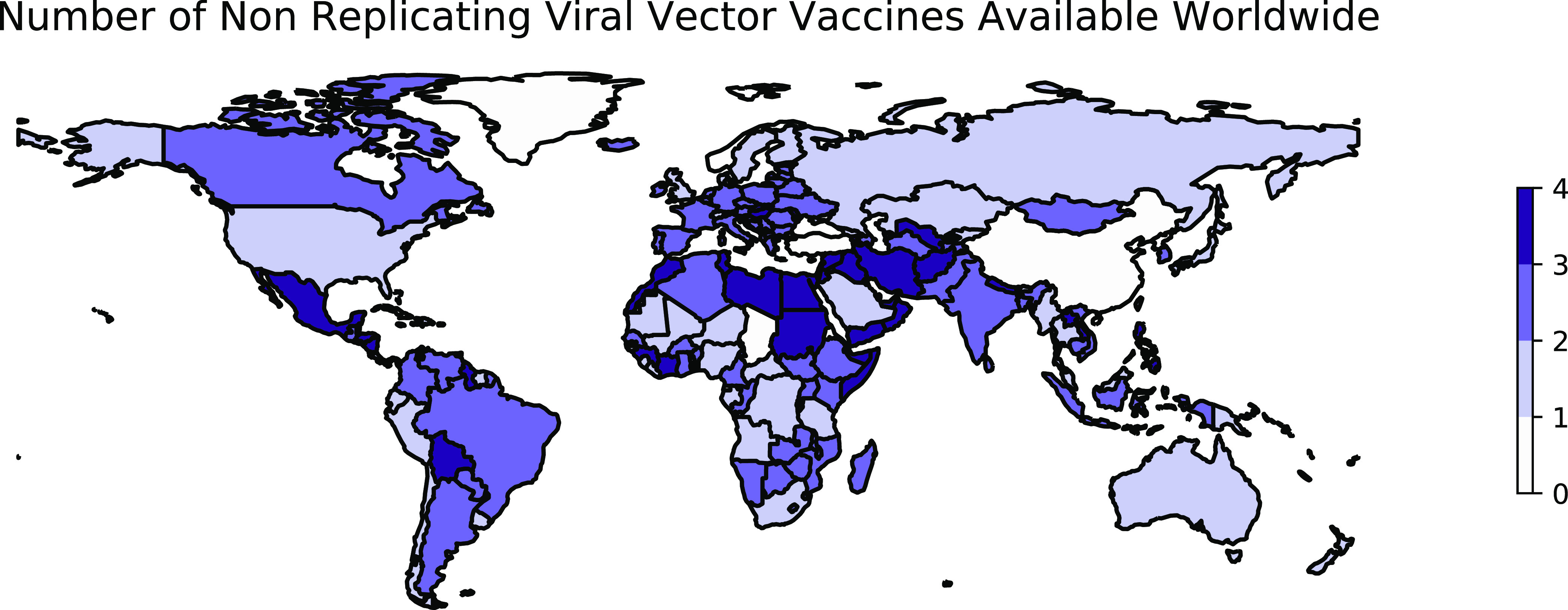
Worldwide availability of vaccines developed using nonreplicating viral vectors. This figure reflects the number of vaccines using nonreplicating viral vectors that were available in each country as of 9 October 2022. These data were retrieved from Our World in Data (70) and plotted using geopandas (236). See https://greenelab.github.io/covid19-review/ for the most recent version of this figure, which is updated daily. Note that this figure draws from a different data source than Table 1 and does not necessarily include data for every vaccine developed within this category.

**(ii) Application to COVID-19.** While not all of the above-described results were available at the time that vaccine development programs against SARS-CoV-2 began, at least three viral vector vaccines have also been developed against SARS-CoV-2 (Fig. 2). First, a collaboration between AstraZeneca and researchers at the University of Oxford successfully applied a viral vector approach to the development of a vaccine against SARS-CoV-2 using the replication-deficient ChAdOx1 vector modified to encode the S protein of SARS-CoV-2 (106). In a phase I trial, the immunogenic potential of vaccine candidate ChAdOx1 nCoV-19 was demonstrated through the immune challenge of two animal models, mice and rhesus macaques (106). In a phase I/II trial, patients receiving the ChAdOx1 nCoV-19 vaccine developed antibodies to the SARS-CoV-2 spike protein that peaked by day 28, with these levels remaining stable until a second observation at day 56 (107).

Second, a viral vector approach was applied by Russia’s Gamaleya Research Institute of Epidemiology and Microbiology to develop Sputnik V, a replication-deficient recombinant adenovirus (rAd) vaccine that combines two adenovirus vectors, rAd26-S and rAd5-S, that express the full-length SARS-CoV-2 spike glycoprotein. These vectors are intramuscularly administered individually using two separate vaccines in a prime-boost regimen. rAd26-S is administered first, followed by rAd5-S 21 days later. Both vaccines deliver 10^11^ viral particles per dose. This approach is designed to overcome any potential preexisting immunity to adenovirus in the population (108), as some individuals may possess immunity to Ad5 (109). Sputnik V is the only recombinant adenovirus vaccine to utilize two vectors.

Third, Janssen Pharmaceuticals, Inc., a subsidiary of Johnson & Johnson, developed a viral vector vaccine in collaboration with and funded by the United States’ Operation Warp Speed (110, 111). The vaccine candidate JNJ-78436735, formerly known as Ad26.COV2-S, is a monovalent vaccine that is composed of a replication-deficient adenovirus serotype 26 (Ad26) vector expressing the stabilized prefusion S protein of SARS-CoV-2 (33, 112). Unlike the other two viral vector vaccines available to date, JNJ-78436735 requires only a single dose, a characteristic that was expected to aid in global deployment (113). JNJ-78436735 was selected from among a number of initial candidate designs (33) and tested *in vivo* in Syrian golden hamsters and rhesus macaques to assess safety and immunogenicity (33, 113–115). The JNJ-78436735 candidate was selected for its favorable immunogenicity profile and ease of manufacturability (33, 113–115) and was found to confer protection against SARS-CoV-2 in macaques even after 6 months (116). The one- versus two-dose regimen was then tested in volunteers through a phase I/IIa trial (112, 117). A major difference between this vaccine and the other two in this category is that the S protein immunogen is stabilized in its prefusion conformation, while in the Sputnik V and AstraZeneca vaccines it is not.

As of 9 October 2022, data describing the distribution of 4 virus-vectored vaccines in 201 countries are available (Fig. 2). ChAdOx1 nCoV-19 was first approved for emergency use on 30 December 2020 in the United Kingdom (118). Sputnik V was available soon after, and as early as January 2021, Sputnik V had been administered to 1.5 million Russians (119) and distribution of doses to other countries within Europe, such as Belarus, Bosnia-Herzegovina, Hungary, San Marino, Serbia, and Slovakia, was begun (120–122).

**(iii) Trial estimates of safety and efficacy.** The first DNA virus-vectored vaccine for which efficacy estimates became available was AstraZeneca’s ChAdOx1 nCoV-19. In December 2020, preliminary results of the phase III trial were released detailing randomized control trials conducted in the United Kingdom, Brazil, and South Africa between April and November 2020 (12). These trials compared ChAdOx1 nCoV-19 to a control, but the design of each study varied; pooling data across studies indicated an overall efficacy of 70.4%. For Sputnik V, the phase III trial indicated an overall vaccine efficacy of 91.6% for symptomatic COVID-19 (123). As for Janssen, the vaccine was well tolerated, and across all regions studied, it was found to be 66.9% effective after 28 days for the prevention of moderate to severe COVID-19 and to be 81.7% effective for the prevention of laboratory-confirmed severe COVID-19 (124). There were no COVID-19-associated deaths in the vaccine group. However, the emergence of the Beta variant in the South African trial population was associated with a slightly reduced efficacy (64% 2 weeks after receipt), and all of the COVID-19-associated deaths in the trial occurred in the South African placebo cohort (124). In February 2021, the FDA issued an emergency use authorization (EUA) for the Janssen vaccine based on interim results from the phase III trial (125, 126).

Two of the three vaccines have faced a number of criticisms surrounding the implementation of their clinical trials. In the race to develop vaccines against SARS-CoV-2, President Vladimir Putin of Russia announced the approval of the Sputnik V vaccines on 11 August 2020 in the absence of clinical evidence (127). A press release on 11 November 2020 indicated positive results from an interim analysis of the phase III Sputnik V trials, which reported 92% efficacy in 16,000 volunteers (128). However, this release came only 2 days after both Pfizer and BioNTech reported that their vaccines had an efficacy over 90%, which led to significant skepticism of the Russian findings for myriad reasons, including the lack of a published protocol and the “reckless” approval of the vaccine in Russia months prior to the publication of the interim results of the phase III trial (128, 129). Consequently, many international scientific agencies and public health bodies expressed concern that due diligence to the clinical trial process was subverted for the sake of expediency, leading many to question the safety and efficacy of Sputnik V (127, 130, 131). Despite regulatory, safety, and efficacy concerns, preorders for 1 billion doses of the Sputnik V were reported within days of the vaccine’s approval in Russia (127). Almost a month later, the phase I/II trial data were published (132). It was not until February 2021, 6 months after its approval in Russia, that interim results of the phase III trial were released (123). This publication reported a VE of 91% and a low rate of serious AEs, although there were several serious AEs that were determined not to be associated with the vaccine by an independent data monitoring committee about which little other information was released (133).

AstraZeneca’s clinical trial also faced criticism. The trial was paused in September 2020 following a severe adverse event in one participant (134). It was restarted soon after (135), but it seems that the recent pause was not mentioned to the FDA during a call the morning before the story broke (136). Additionally, individual sites within the trial employed somewhat different designs but were combined for analysis. For example, in South Africa, the trial was double-blinded, whereas in the United Kingdom and Brazil it was single-blinded, and one of the two trials carried out in the United Kingdom evaluated two dosing regimens (low dose and standard dose, both followed by standard dose). Some of the trials used a meningococcal conjugate vaccine (MenACWY) as a control, while others used saline. Data were pooled across countries for analysis, a design decision that was approved by regulators but raised some questions when higher efficacy was reported in a subgroup of patients who received a low dose followed by a standard dose. This group came about because some participants in the United Kingdom were erroneously primed with a much lower dose, which turned out to have higher efficacy than the intended dose (137). Combining the data then led to confusion surrounding the VE, as VE varied widely among conditions (e.g., 62% VE in the standard dose group versus 90% in the group that received a low prime dose [12]). Subsequent research, however, suggests that reducing the prime dose may, in fact, elicit a superior immune response in the long term despite a lower initial response (138). Therefore, this error may serendipitously improve efficacy of vaccine-vectored vaccines broadly.

**(iv) Real-world safety and efficacy.** Following the trials, additional concerns have been raised about some of these vaccines. Within a few days to a few weeks following their first dose of the AstraZeneca vaccine, three women developed extensive venous sinus thrombosis (139). In March 2021, administration of the vaccine was paused in several European countries while a possible link to thrombotic events was investigated (140), as these adverse events had not been observed in clinical trials, but the European Medicines Agency (EMA) soon determined that 25 events were not related to the vaccine (141). The following month, the United States paused administration of the Janssen vaccine for 10 days due to 15 similar AEs (142, 143), but the EMA, U.S. Centers for Disease Control, and the FDA’s Advisory Committee on Immunization Practices again identified the events as being very rare and the benefits of the vaccine as likely to outweigh its risks (144–147). In Denmark and Norway, population-based estimates suggested that AstraZeneca’s vaccine increased the incidence of venous thromboembolic events by 11 cases over baseline per 100,000 doses (148). Estimates of the incidence in other Western countries have also been low (149). In the United States, thromboembolic events following the Janssen vaccine have also been very rare (145). Subsequently, a potential mechanism was identified: the adenovirus vector binding to platelet factor 4 (150, 151). Because this adverse event is so rare, the risk is likely still outweighed by the risks associated with contracting COVID-19 (152), which is also associated with thrombotic events (143, 153). Similarly, concerns about Guillain-Barré syndrome arose in connection to the Janssen vaccine, but these events have similarly been determined to be very rare and the benefits to outweigh the risks (147).

Given that vaccines from multiple platforms are now widely available, people at increased risk of a specific severe AE may have options to pursue vaccination with a platform that does not carry such risks. For example, a woman in the United States with a history of thromboembolic concerns might feel more comfortable with an mRNA vaccine (described below), because such AEs have not been identified in association with COVID-19 vaccination in the United States. However, within the United States, no clear framework has been established for advising patients on whether a specific vaccine may be preferable for their individual concerns now that vaccines based on three different technologies are widely available (see reference 1 for information about Novavax, which is a protein subunit vaccine).

## mRNA VACCINES

Building on DNA vaccine technology, RNA vaccines are an even more recent advancement for vaccine development. Interest in mRNA vaccines emerged around 1990 following *in vitro* and animal model studies that demonstrated that mRNA could be transferred into cells (154, 155). mRNA contains the minimum information needed to create a protein (155). RNA vaccines are therefore nucleic acid-based modalities that code for viral antigens against which the human body elicits humoral and cellular immune responses.

The strategy behind mRNA vaccines operates one level above the DNA: instead of directly furnishing the gene sequence associated with an antigen to the host, it provides the mRNA transcribed from the DNA sequence. The mRNA is transcribed *in vitro* and delivered to cells via lipid nanoparticles (LNPs) (156). It is recognized by ribosomes *in vivo* and then translated and modified into functional proteins (157). The resulting intracellular viral proteins are displayed on surface MHC proteins, provoking a strong CD8^+^ T cell response as well as CD4^+^ T cell- and B cell-associated antibody responses (157). mRNA is naturally not very stable and can degrade quickly in the extracellular environment or the cytoplasm. The LNP covering protects the mRNA from enzymatic degradation outside of the cell (158). Codon optimization to prevent secondary-structure formation and modifications of the poly(A) tail as well as the 5′ untranslated region (UTR) to promote ribosomal complex binding can increase mRNA expression in cells. Furthermore, purifying out double-stranded RNA and immature RNA with fast-performance liquid chromatography and high-performance liquid chromatography technology improves translation of the mRNA in the cell (157, 159).

There are three types of RNA vaccines: nonreplicating, *in vivo* self-replicating, and *in vitro* dendritic cell nonreplicating (160). Nonreplicating mRNA vaccines consist of a simple open reading frame for the viral antigen flanked by the 5′ UTR and 3′ poly(A) tail. *In vivo* self-replicating vaccines encode a modified viral genome derived from single-stranded, positive-sense RNA alphaviruses (157, 159). The RNA genome encodes the viral antigen along with proteins of the genome replication machinery, including an RNA polymerase. Structural proteins required for viral assembly are not included in the engineered genome (157). Self-replicating vaccines produce more viral antigens over a longer period of time, thereby evoking a more robust immune response (160). Finally, *in vitro* dendritic cell nonreplicating RNA vaccines limit transfection to dendritic cells. Dendritic cells are potent antigen-presenting immune cells that easily take up mRNA and present fragments of the translated peptide on their MHC proteins, which can then interact with T cell receptors. Ultimately, primed T follicular helper cells can stimulate germinal center B cells that also present the viral antigen to produce antibodies against the virus (161). These cells are isolated from the patient and then grown and transfected *ex vivo* (162). They can then be reintroduced to the patient (162).

In addition to the benefits of nucleic acid vaccines broadly, mRNA confers specific advantages compared to DNA vaccines and other platforms (163). Some of these advantages fall within the domain of safety. Unlike DNA vaccines, mRNA technologies are naturally degradable and nonintegrating, and they do not need to cross the nuclear membrane in addition to the plasma membrane for their effects to be seen (157). Additionally, the half-life can be regulated by the contents of the 5′ and 3′ untranslated regions (164). In comparison to vaccines that use live attenuated viruses, mRNA vaccines are noninfectious and can be synthetically produced in an egg-free, cell-free environment, thereby reducing the risk of a detrimental immune response in the host (165). Furthermore, mRNA vaccines are easily, affordably, and rapidly scalable, despite the fact that it took time to reach the scale needed to manufacture vaccines sufficient for the global population (163).

### Prior applications.

Although mRNA vaccines have been developed for therapeutic and prophylactic purposes, none have previously been licensed or made commercially available. Challenges were caused by the instability of mRNA molecules, the design requirements of an efficient delivery system, and the potential for mRNA either to elicit a very strong immune response or to stimulate the immune system in secondary ways (17, 166). As of the 2010s, mRNA was still considered a promising technology for future advances in vaccine development (155), but prior to 2020, no mRNA vaccines had been approved for use in humans, despite significant advances in the development of this technology (162). This approach showed promise in animal models and preliminary clinical trials for several indications, including rabies, coronavirus, influenza, and cytomegalovirus (167). Preclinical data previously identified effective antibody generation against full-length purified influenza virus hemagglutinin stalk-encoding mRNA in mice, rabbits, and ferrets (168). Similar immunological responses for mRNA vaccines were observed in humans in phase I and II clinical trials operated by the pharmaceutical-development companies Curevac and Moderna for rabies, flu, and Zika (159). Positively charged bilayer LNPs carrying the mRNA attract negatively charged cell membranes, endocytose into the cytoplasm (158), and facilitate endosomal escape. LNPs can be coated with modalities recognized and engulfed by specific cell types, and LNPs that are 150 nm or less effectively enter into lymphatic vessels (158, 169). Therefore, while these technologies elegantly capitalize on decades of research in vaccine development as well as the tools of the genomic revolution, it was largely unknown prior to the SARS-CoV-2 pandemic whether this potential could be realized in a real-world vaccination effort.

### Application to COVID-19.

Given the potential for mRNA technology to be quickly adapted for a new pathogen, it was favored as a potential vaccine against COVID-19, and fortunately, the prior work in mRNA vaccine development paid off, with 8 mRNA vaccines available in at least one country as of 7 October 2022 (Table 2). In the vaccines developed under this approach, the mRNA coding for a stabilized prefusion spike protein, which is immunogenic (170), is furnished to the immune system in order to train its response.

**TABLE 2:** mRNA vaccines approved in at least one country (237) as of 7 October 2022^*a*^

Vaccine	Company
GEMCOVAC-19	Gennova Biopharmaceuticals Limited
Spikevax	Moderna
Spikevax Bivalent Original/Omicron BA.1	Moderna
Spikevax Bivalent Original/Omicron BA.4/BA.5	Moderna
Comirnaty	Pfizer/BioNTech
Comirnaty Bivalent Original/Omicron BA.1	Pfizer/BioNTech
Comirnaty Bivalent Original/Omicron BA.4/BA.5	Pfizer/BioNTech
TAK-919 (Moderna formulation)	Takeda

a This table includes licensing of existing mRNA technology; i.e., TAK-919 is used to describe Takeda’s manufacturing of Moderna’s formulation.

**FIG 3:**
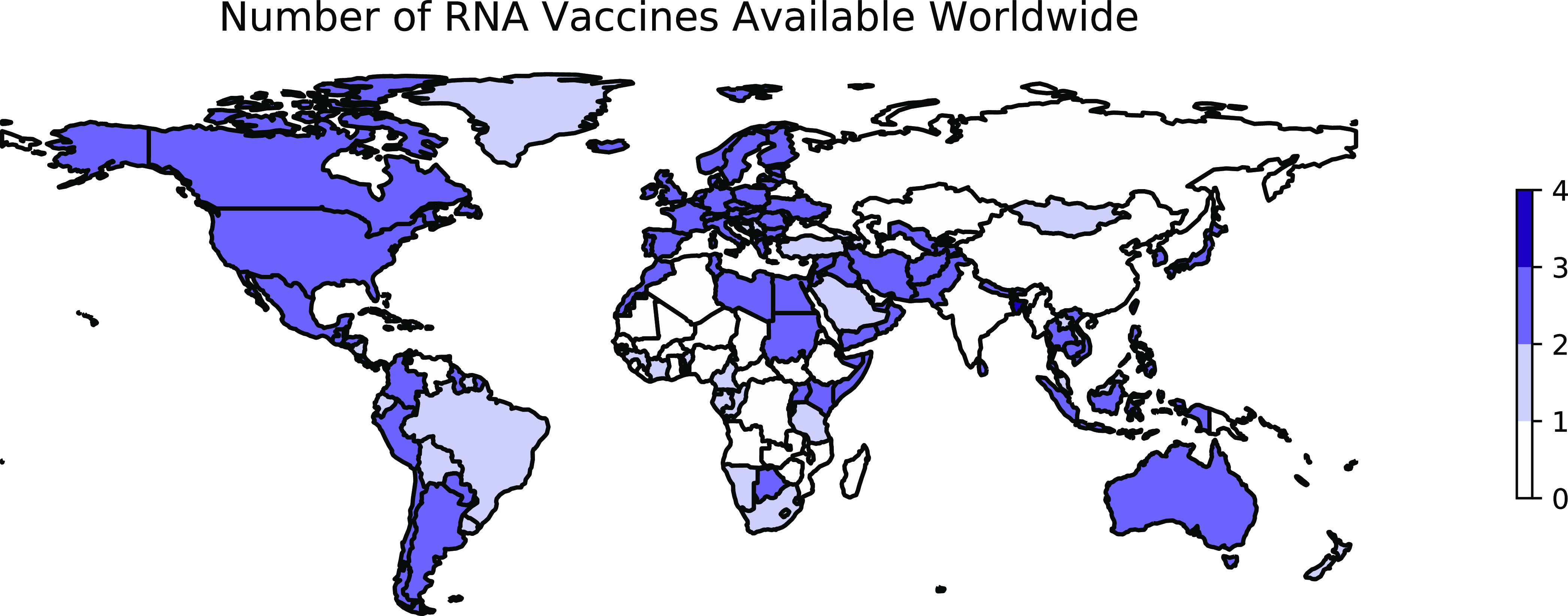
Worldwide availability of vaccines developed using mRNA. This figure reflects the number of vaccines based on mRNA technology that were available in each country as of 9 October 2022. These data are retrieved from Our World in Data (70) and plotted using geopandas (236). See https://greenelab.github.io/covid19-review/ for the most recent version of this figure, which is updated daily. Note that this figure draws from a different data source than Table 2 and does not necessarily include data for every vaccine developed within this category.

Two vaccine candidates in this category emerged with promising phase III results at the end of 2020. Both require two doses approximately 1 month apart. The first was Pfizer/BioNTech’s BNT162b2, which contains the full prefusion stabilized, membrane-anchored SARS-CoV-2 spike protein in a vaccine formulation based on modified mRNA (modRNA) technology (171, 172). The second mRNA vaccine, mRNA-1273 developed by ModernaTX, is comprised by a conventional LNP-encapsulated RNA encoding a full-length prefusion stabilized S protein for SARS-CoV-2 (173). The vaccine candidates developed against SARS-CoV-2 using mRNA vectors utilize similar principles and technologies, although there are slight differences in implementation among candidates, such as the formulation of the platform and the specific components of the spike protein encapsulated (e.g., the full spike protein versus the receptor binding domain alone) (174). As of 9 October 2022, 2 mRNA vaccines are available in 169 countries (Fig. 3).

The rapid and simultaneous development of these vaccines was met with some controversy related to intellectual property (IP). First, the National Institutes of Health (NIH) and Moderna became involved in a patent dispute, after researchers at the NIH argued that they were unfairly excluded from some patents filed based on their IP after they generated the stabilized modRNA sequence used in the vaccine (175). Ultimately, in late 2021, Moderna backed down on the patent application (176). However, in August 2022, the company filed their own suit against Pfizer/BioNTech over IP related to the modRNA used in the latter’s COVID-19 vaccine (176, 177). The outcome of this suit remains to be seen.

### Trial safety and immunogenicity.

The VEs revealed by the Pfizer/BioNTech and Moderna clinical trials exceeded expectations. In a phase II/III multinational trial, Pfizer/BioNTech’s BNT162b2 vaccine was associated with a 95% efficacy against laboratory-confirmed COVID-19 and with mild to moderate local and systemic effects but a low risk of serious AEs when the prime-boost doses were administered 21 days apart (178). The ModernaTX mRNA-1273 vaccine was the second mRNA vaccine for which phase III results were released, despite being the first mRNA vaccine to enter phase I clinical trials and have interim results of the phase III trial published a few months later. That study reported a 94.5% vaccine efficacy in preventing symptomatic COVID-19 in adults who received the vaccine at 99 sites around the United States (179). Similar to BNT162b2, the mRNA-1273 vaccine was associated with mild to moderate AEs but with a low risk of serious AEs (179). In late 2020, both vaccines received approval from the FDA under an emergency use authorization (180, 181), and these vaccines have been widely distributed, primarily in North America and the European Union (182). As the first mRNA vaccines to make it to market, these two highly efficacious vaccines demonstrate the power of this emerging technology, which has previously attracted scientific interest because of its potential to be used to treat noninfectious as well as infectious diseases.

### Real-world safety and effectiveness.

As vaccines were rolled out, one study sought to monitor their effectiveness in a real-world setting. Between December 2020 and April 2021, this prospective cohort study obtained weekly nasal swabs from 3,975 individuals at high risk of SARS-CoV-2 exposure (health care workers, frontline workers, etc.) within the United States (183). Among these participants, 3,179 (80%) had received at least one dose of an mRNA vaccine, and of those, 2,686 (84%) were fully vaccinated, corresponding to 68% of trial participants overall. For each vaccinated participant (defined here as having received at least dose 1 more than 7 days ago) whose sample tested positive for SARS-CoV-2, the investigators categorized the viral lineage(s) present in the sample as well as in samples from 3 or 4 unvaccinated individuals matched by site and testing date. The overall efficacy of mRNA vaccines was estimated at 91% with full vaccination, similar to the reports from the clinical trials. The occurrence of fevers was also lower in individuals who were partially or fully vaccinated, and the duration of symptoms was approximately 6 days shorter. Among the 5 cases in fully vaccinated and 11 cases in partially vaccinated participants, the rate of infection by VOC was much higher than in the unvaccinated population (30% versus 10%), suggesting that the vaccine was less effective against the VOC than the index strain.

The WHO continues to monitor the emergence of variants and their impact on vaccine efficacy (184). In general, mRNA vaccines remain highly effective against severe illness and death, but the effectiveness against infection generally has declined. A study monitoring infections in a Minnesota cohort from January to July 2021 estimated that the effectiveness of the Moderna vaccine fell to 86% and Pfizer to 76%, although protection against hospitalization remained at 91% and 85%, respectively (185). In July of that year, as the Delta variant became dominant in the United States, these estimates all fell, to an effectiveness of 76% for Moderna and 42% for Pfizer and effectiveness against hospitalization of 81% and 75%, respectively (185).

With the emergence of the Omicron VOC, vaccine effectiveness has likely further declined. A study in a diverse cohort in Southern California, USA, found the effectiveness of the Moderna vaccine in participants who had received only the primary course to be 44% (186). A study in South Africa compared case and hospitalization records from a 4-week period when Omicron was dominant to a 2-month period when Delta was dominant and found that the effectiveness against hospitalization during the Omicron wave was approximately 70%, compared to 93% during the Delta wave (187). Similarly, a large study in England of 2.5 million individuals suggested that not only the variants circulating but also the time since vaccination played a large role in vaccine effectiveness (188). Shortly after the BNT162b2 primary course, effectiveness against the Omicron VOC was as high as 65.5%, but this declined to below 10% by 6 months after the second dose. For mRNA-1273, the decline was from 75.1% to 14.9%. Therefore, it is unsurprising that in spite of vaccination programs, infection rates and hospitalization rates climbed in early 2022 in many Western countries, including the United States (189, 190), especially given that many places simultaneously began to loosen public health restrictions designed to reduce viral spread.

On the side of safety, the only major concern that has been raised is a possible link between mRNA vaccination and myocarditis, especially in young men (147). This concern began with case reports of a small number of cases of myocarditis following vaccination in several countries (191, 192). Following these reports, the Israeli Ministry of Health began surveillance to monitor the occurrence of myocarditis (193). They identified 283 cases, almost exactly half of which occurred following vaccination with Pfizer’s BNT162b2. Close analysis of these cases determined that the vaccine did have a significant effect on the incidence of myocarditis; however, the rate of myocarditis remained low overall (193). The identification of young men as a population at particular risk of this AE was supported, and the risk was found to be greater after the second dose than the first. Both this study and a study evaluating data collected from U.S. population-based surveillance identified an increased risk with additional doses (194). However, most findings suggest that this AE does not have long-term negative effects; a 2021 meta-analysis identified 69 cases, all of which resulted in full recovery (195). Although these events are very rare, as with the possible thromboembolic AEs associated with virus-vectored DNA vaccines, these findings suggest that it may be prudent to offer a framework for decision making for patients particularly concerned about specific AEs in settings where multiple vaccines are available.

## BOOSTER DOSES

Due to waning effectiveness of vaccines over time, especially in light of viral evolution, boosters have emerged as an important strategy in retaining the benefits of vaccination over time. Booster shots are now recommended in many places, and boosters that account for multiple variants and strains of SARS-CoV-2 are now available in some places (196). For example, in the United States, the FDA recently recommended bivalent booster doses designed to account for the Omicron VOC (197–199). In this case, “bivalent” refers to the fact that doses deliver both the original formulation and an updated vaccine designed for the Omicron subvariants circulating in summer 2022. The fact that the FDA did not require additional clinical trials from manufacturers for Omicron subvariants BA.4 and BA.5 specifically suggests that the rapid authorization of strain changes in response to emerging VOC may be increasingly attainable (200). Results suggest that this fourth dose offered at least a short-term increase in VE against Omicron subvariants and also provided additional protection against hospitalization (201).

Homologous booster doses have been investigated for most vaccines. For example, over 14,000 adults were administered a booster (second) dose of the Janssen Ad26.COV2.S vaccine (202). The booster dose was highly efficacious, with severe COVID-19 and hospitalization prevented almost completely in the vaccinated group. A booster dose was also found to improve immune response for Sputnik V vaccinees (203). For the AstraZeneca vaccine, a different approach was taken. In the interest of distributing first doses as widely as possible, in some places the time between the first and second doses was extended. One study assessed the immunogenicity and reactogenicity associated with delaying the second dose in the prime-boost series until up to 45 weeks after the first, reporting that an extended interdose period was associated with increased antibody titers 28 days after the second dose (204). This analysis also revealed that a third dose provided an additional boost in neutralizing activity (204).

Third and fourth doses have been introduced for at least some populations in many places in response to the Omicron variant. An early study in Israeli health care workers showed that the additional immunization was safe and immunogenic, with antibody titers restored to peak third-dose titers. No severe illness was reported in the cohort studied (274 versus 426 age-matched controls), and vaccine efficacy against infection was reported at 30% for BNT162b2 and 11% for mRNA-1273 (205). Other studies reported that a third dose of BNT162b2 raised vaccine effectiveness to 67.2% for approximately the first month but that the effectiveness dropped to 45.7% (188). Reduced and even low efficacy against infection does not undermine the value of vaccination, considering the vaccines are intended to prevent severe disease, hospitalization, and death rather than infection generally. However, these findings do suggest that boosters will likely be needed as the virus continues to evolve.

Many trials have also investigated heterologous boosting approaches. In particular, the mRNA vaccines are a popular choice for booster doses regardless of primary series. In general, such approaches have been found to confer favorable immunogenicity relative to homologous boosters (for examples, see references 206 to 212). Due to remaining concerns about rare thromboembolic events, vaccinees who received AstraZeneca for their primary course are advised in some countries to seek a heterologous booster (213), although such guidance is not supported by the evidence, which indicates that the first dose of AstraZeneca is most likely to be linked to these rare events (214). In general, heterologous boosting with mRNA vaccines elicits a strong immune response. For patients who received BNT162b2 as a heterologous booster following a ChAdOx1 primary series, the vaccine effectiveness was estimated to be 62.4% initially, dropping to 39.6% after 10 weeks (188). For a heterologous mRNA-1273 booster, the effectiveness was estimated to be slightly higher (70.1% and 60.9% following ChAdOx1 and 73.9% to 64.4% following BNT162b2) (188). Therefore, subsequent booster doses may remain an ongoing component of strategies to combat SARS-CoV-2.

Although the vaccines developed based on the index strain remain highly effective at preventing severe illness and death, they provide much less utility at preventing illness broadly than they did early in the pandemic. Therefore, many manufacturers are exploring potential reformulations based on VOC that have emerged since the beginning of the pandemic. In June 2022, Moderna released data describing the effect of their bivalent mRNA booster, mRNA-1273.214, designed to protect against the Omicron variant (215). A 50-μg dose of mRNA-1273.214 was administered to 437 participants. One month later, the neutralizing geometric mean titer ratio was assessed against several variants of SARS-CoV-2, including Omicron. The immune response was higher against all variants assessed, including Omicron, than for boosting with the original formulation (mRNA-1273). Another formulation, mRNA-1273.211, developed based on the Beta variant, has been associated with durable protection as long as 6 months after dosing. The associated publications suggest that this novel formulation offers significant protection against Omicron and other VOC (216, 217). In August 2022, Pfizer also announced successful development of a new formulation effective against Omicron (218).

Modularity has been proposed as one of the advantages to developing DNA and mRNA vaccines. This design would allow for faster adaptation to viral evolution. However, in the arms race against SARS-CoV-2, the vaccines are still lagging behind the virus. This disadvantage may change as regulators become more familiar with these vaccines and as a critical mass of data is accumulated. Given the apparent need for boosters, interest has also emerged in whether updated formulations of SARS-CoV-2 vaccines can be administered along with annual flu vaccines to improve immunity to novel variants.

## CONCLUSIONS

COVID-19 has seen the coming of age of vaccine technologies that have been in development since the late 20th century but had never before been authorized for use. Vaccines that employ DNA and RNA eliminate all concerns about potential infection due to the vaccine components. The vaccines described above demonstrate the potential for these technologies to facilitate a quick response to an emerging pathogen. Additionally, their efficacy in trials far exceeded expectations, especially in the case of RNA vaccines. These technologies hold significant potential to drive improvements in human health over the coming years.

Traditional vaccine technologies were built on the principle of using either a weakened version of the virus or a fragment of the virus. COVID-19 has highlighted the fact that in recent years, the field has undergone a paradigm shift toward reverse vaccinology. Reverse vaccinology emphasizes a discovery-driven approach to vaccine development based on knowledge of the viral genome (219). This strategy was explored during development of a DNA vaccine against the Zika virus (220). Though the disease was controlled before the vaccine became available (2), the response demonstrated the potential for modular technologies to facilitate a response to emerging viral threats (220). The potential for such vaccines to benefit the field of oncology has encouraged vaccine developers to invest in next-generation approaches, which has spurred the diversification of vaccine development programs (22, 221). As a result, during the COVID-19 pandemic, these modular technologies have taken center stage in controlling a viral threat for the first time.

The safety and efficacy of vaccines that use these new technologies have exceeded expectations. While there were rare reports of severe AEs such as myocarditis (mRNA platforms) and thromboembolic events (virus-vectored DNA platforms), widespread availability of both types of vaccines would allow individuals to choose (particularly relevant in this case because myocarditis has primarily been reported for men and thromboembolic events primarily for women). Estimates of efficacy have varied widely but in all cases are high. Estimates of the efficacy of DNA vaccine platforms have typically fallen in the range of approximately either 67% (ZyCoV-D and Janssen) or 90% (Sputnik V). AstraZeneca’s trial produced estimates in both ranges, with the standard dosage producing an efficacy of 62% and the lower prime dose producing a VE of 90%. The mRNA vaccine trials were somewhat higher, with VE estimated at approximately 95% for both the Moderna and Pfizer/BioNTech clinical trials. However, in all cases, the efficacies against severe illness and death were very high. Therefore, all of these vaccines are useful tools for combating COVID-19.

Furthermore, the fact that vaccine efficacy is not a static value has become particularly salient, as real-world effectiveness has changed with location and over time. COVID-19 vaccines have been challenged by the emergence of VOC. These VOC generally carry genetic mutations that code for an altered spike protein (i.e., the antigen), so the antibodies resulting from immunization with vaccines developed from the index strain neutralize them less effectively (222, 223). Despite some reports of varying and reduced effectiveness or efficacy of the mRNA vaccines against the Alpha (B.1.1.7), Beta (B.1.351), and Delta (B.1.617.2) variants versus the original SARS-CoV-2 strain or the D614G variant (224–226), the greatest concern to date has been the Omicron variant (B.1.1.529), which was first identified in November 2021 (223, 227). As of March 2022, the Omicron variant accounted for 95% of all infections sequenced in the United States (228) and was linked to an increased risk of SARS-CoV-2 reinfection (222) and further infection of those who have been vaccinated with the mRNA vaccines (229).

One of the downsides of this leap in vaccine technologies, however, is that they have largely been developed by wealthy countries, including countries in the European Union, the United States, the United Kingdom, and Russia. As a result, they are also largely available to residents of wealthy countries, primarily in Europe and North America. Although the VE of DNA vaccines tends to be lower than that of mRNA vaccines (230), they still provide excellent protection against severe illness and are much easier to distribute due to less complex demands for storage. Efforts, such as COVID-19 Vaccines Global Access (COVAX), that aim to expand access to vaccines developed by wealthy countries have not been as successful as hoped (231). Fortunately, vaccine development programs using more established technologies have been undertaken in many middle-income countries, and those vaccines have been more accessible globally (1). Additionally, efforts to develop new formulations of DNA vaccines in lower- and middle-income countries are increasingly being undertaken (232).

The modular nature of nucleic acid-based vaccine platforms has opened a new frontier in responding to emerging viral illnesses. The RNA vaccines received an EUA in only a few months more than it took to identify the pathogen causing SARS in 2002. Given the variety of options available for preventing severe illness and death, it is possible that certain vaccines may be preferable for certain demographics (e.g., young women might choose an mRNA vaccine to entirely mitigate the very low risk of blood clots [147]). However, this option is likely only available to people in high-income countries. In lower-income countries, access to vaccines broadly is a more critical issue. Different vaccines may confer advantages in different countries, and vaccine development in a variety of cultural contexts is therefore important (233). Without widespread access to vaccines on the global scale, SARS-CoV-2 will continue evolving, presenting a threat to all nations.

## References

[1] Rando HM, Lordan R, Lee AJ, Naik A, Wellhausen N, Sell E, Kolla L, COVID-19 Review Consortium, Gitter A, Greene CS. 2023. Application of traditional vaccine development strategies to SARS-CoV-2. mSystems. doi:10.1128/mSystems.00927-22. PMC10134813 36861991

[2] Lurie N, Saville M, Hatchett R, Halton J. 2020. Developing Covid-19 vaccines at pandemic speed. N Engl J Med 382:1969–1973. doi:10.1056/NEJMp2005630. 32227757

[3] Graham RL, Donaldson EF, Baric RS. 2013. A decade after SARS: strategies for controlling emerging coronaviruses. Nat Rev Microbiol 11:836–848. doi:10.1038/nrmicro3143. 24217413 PMC5147543

[4] Cohen J. 2014. Ebola vaccine: little and late. Science 345:1441–1442. doi:10.1126/science.345.6203.1441. 25237082

[5] Coller B-AG, Blue J, Das R, Dubey S, Finelli L, Gupta S, Helmond F, Grant-Klein RJ, Liu K, Simon J, Troth S, VanRheenen S, Waterbury J, Wivel A, Wolf J, Heppner DG, Kemp T, Nichols R, Monath TP. 2017. Clinical development of a recombinant Ebola vaccine in the midst of an unprecedented epidemic. Vaccine 35:4465–4469. doi:10.1016/j.vaccine.2017.05.097. 28647166

[6] Rando HM, Wellhausen N, Ghosh S, Lee AJ, Dattoli AA, Hu F, Byrd JB, Rafizadeh DN, Lordan R, Qi Y, Sun Y, Brueffer C, Field JM, Ben Guebila M, Jadavji NM, Skelly AN, Ramsundar B, Wang J, Goel RR, Park Y, Bansal V, Barton JP, Boca SM, Boerckel JD, Brueffer C, Byrd JB, Capone S, Das S, Dattoli AA, Dziak JJ, Field JM, Ghosh S, Gitter A, Goel RR, Greene CS, Guebila MB, Himmelstein DS, Hu F, Jadavji NM, Kamil JP, Knyazev S, Kolla L, Lee AJ, Lordan R, Lubiana T, Lukan T, MacLean AL, Mai D, Mangul S, Manheim D, COVID-19 Review Consortium, et al. 2021. Identification and development of therapeutics for COVID-19. mSystems 6:e00233-21. doi:10.1128/mSystems.00233-21. 34726496 PMC8562484

[7] Rando HM, MacLean AL, Lee AJ, Lordan R, Ray S, Bansal V, Skelly AN, Sell E, Dziak JJ, Shinholster L, D’Agostino McGowan L, Ben Guebila M, Wellhausen N, Knyazev S, Boca SM, Capone S, Qi Y, Park Y, Mai D, Sun Y, Boerckel JD, Brueffer C, Byrd JB, Kamil JP, Wang J, Velazquez R, Szeto GL, Barton JP, Goel RR, Mangul S, Lubiana T, Bansal V, Barton JP, Boca SM, Boerckel JD, Brueffer C, Byrd JB, Capone S, Das S, Dattoli AA, Dziak JJ, Field JM, Ghosh S, Gitter A, Goel RR, Greene CS, Guebila MB, Himmelstein DS, Hu F, Jadavji NM, COVID-19 Review Consortium, et al. 2021. Pathogenesis, symptomatology, and transmission of SARS-CoV-2 through analysis of viral genomics and structure. mSystems 6:e00095-21. doi:10.1128/mSystems.00095-21. 34698547 PMC8547481

[8] Moderna. 2023. Our story. https://www.modernatx.com/en-US/about-us/our-story?slug=about-us%2Four-story. Accessed 10 October 2022.

[9] Thanh Le T, Andreadakis Z, Kumar A, Gómez Román R, Tollefsen S, Saville M, Mayhew S. 2020. The COVID-19 vaccine development landscape. Nat Rev Drug Discov 19:305–306. doi:10.1038/d41573-020-00073-5. 32273591

[10] Krammer F. 2020. SARS-CoV-2 vaccines in development. Nature 586:516–527. doi:10.1038/s41586-020-2798-3. 32967006

[11] World Health Organization. 2020. Novel coronavirus—China. https://www.who.int/emergencies/disease-outbreak-news/item/2020-DON233. Accessed 10 October 2022.

[12] Voysey M, Clemens SAC, Madhi SA, Weckx LY, Folegatti PM, Aley PK, Angus B, Baillie VL, Barnabas SL, Bhorat QE, Bibi S, Briner C, Cicconi P, Collins AM, Colin-Jones R, Cutland CL, Darton TC, Dheda K, Duncan CJA, Emary KRW, Ewer KJ, Fairlie L, Faust SN, Feng S, Ferreira DM, Finn A, Goodman AL, Green CM, Green CA, Heath PT, Hill C, Hill H, Hirsch I, Hodgson SHC, Izu A, Jackson S, Jenkin D, Joe CCD, Kerridge S, Koen A, Kwatra G, Lazarus R, Lawrie AM, Lelliott A, Libri V, Lillie PJ, Mallory R, Mendes AVA, Milan EP, Minassian AM, et al. 2021. Safety and efficacy of the ChAdOx1 nCoV-19 vaccine (AZD1222) against SARS-CoV-2: an interim analysis of four randomised controlled trials in Brazil, South Africa, and the UK. Lancet 397:99–111. doi:10.1016/S0140-6736(20)32661-1. 33306989 PMC7723445

[13] Duan L, Zheng Q, Zhang H, Niu Y, Lou Y, Wang H. 2020. The SARS-CoV-2 spike glycoprotein biosynthesis, structure, function, and antigenicity: implications for the design of spike-based vaccine immunogens. Front Immunol 11:576622. doi:10.3389/fimmu.2020.576622. 33117378 PMC7575906

[14] Jackson LA, Anderson EJ, Rouphael NG, Roberts PC, Makhene M, Coler RN, McCullough MP, Chappell JD, Denison MR, Stevens LJ, Pruijssers AJ, McDermott A, Flach B, Doria-Rose NA, Corbett KS, Morabito KM, O’Dell S, Schmidt SD, Swanson PA, Padilla M, Mascola JR, Neuzil KM, Bennett H, Sun W, Peters E, Makowski M, Albert J, Cross K, Buchanan W, Pikaart-Tautges R, Ledgerwood JE, Graham BS, Beigel JH, mRNA-1273 Study Group. 2020. An mRNA vaccine against SARS-CoV-2—preliminary report. N Engl J Med 383:1920–1931. doi:10.1056/NEJMoa2022483. 32663912 PMC7377258

[15] Rhee JH. 2014. Towards Vaccine 3.0: new era opened in vaccine research and industry. Clin Exp Vaccine Res 3:1–4. doi:10.7774/cevr.2014.3.1.1. 24427757 PMC3890443

[16] Seib KL, Zhao X, Rappuoli R. 2012. Developing vaccines in the era of genomics: a decade of reverse vaccinology. Clin Microbiol Infect 18:109–116. doi:10.1111/j.1469-0691.2012.03939.x. 22882709

[17] Liu MA. 2019. A comparison of plasmid DNA and mRNA as vaccine technologies. Vaccines (Basel) 7:37. doi:10.3390/vaccines7020037. 31022829 PMC6631684

[18] Plotkin S. 2014. History of vaccination. Proc Natl Acad Sci USA 111:12283–12287. doi:10.1073/pnas.1400472111. 25136134 PMC4151719

[19] Cui Z. 2005. DNA vaccine. Adv Genet 54:257–289. doi:10.1016/S0065-2660(05)54011-2. 16096015 PMC7119308

[20] Ellis RW, Douglas RG. 1994. New vaccine technologies. JAMA 271:929–931. doi:10.1001/jama.1994.03510360055036. 8120962

[21] Liu MA. 2003. DNA vaccines: a review. J Intern Med 253:402–410. doi:10.1046/j.1365-2796.2003.01140.x. 12653868

[22] Kutzler MA, Weiner DB. 2008. DNA vaccines: ready for prime time? Nat Rev Genet 9:776–788. doi:10.1038/nrg2432. 18781156 PMC4317294

[23] Sternberg A, Naujokat C. 2020. Structural features of coronavirus SARS-CoV-2 spike protein: targets for vaccination. Life Sci 257:118056. doi:10.1016/j.lfs.2020.118056. 32645344 PMC7336130

[24] Li F. 2016. Structure, function, and evolution of coronavirus spike proteins. Ann Rev Virol 3:237–261. doi:10.1146/annurev-virology-110615-042301. 27578435 PMC5457962

[25] Kirchdoerfer RN, Cottrell CA, Wang N, Pallesen J, Yassine HM, Turner HL, Corbett KS, Graham BS, McLellan JS, Ward AB. 2016. Pre-fusion structure of a human coronavirus spike protein. Nature 531:118–121. doi:10.1038/nature17200. 26935699 PMC4860016

[26] Walls AC, Park Y-J, Tortorici MA, Wall A, McGuire AT, Veesler D. 2020. Structure, function, and antigenicity of the SARS-CoV-2 spike glycoprotein. Cell 181:281–292.e6. doi:10.1016/j.cell.2020.02.058. 32155444 PMC7102599

[27] Liu C, Mendonça L, Yang Y, Gao Y, Shen C, Liu J, Ni T, Ju B, Liu C, Tang X, Wei J, Ma X, Zhu Y, Liu W, Xu S, Liu Y, Yuan J, Wu J, Liu Z, Zhang Z, Liu L, Wang P, Zhang P. 2020. The architecture of inactivated SARS-CoV-2 with postfusion spikes revealed by cryo-EM and cryo-ET. Structure 28:1218–1224.e4. doi:10.1016/j.str.2020.10.001. 33058760 PMC7557167

[28] Ke Z, Oton J, Qu K, Cortese M, Zila V, McKeane L, Nakane T, Zivanov J, Neufeldt CJ, Cerikan B, Lu JM, Peukes J, Xiong X, Kräusslich H-G, Scheres SHW, Bartenschlager R, Briggs JAG. 2020. Structures and distributions of SARS-CoV-2 spike proteins on intact virions. Nature 588:498–502. doi:10.1038/s41586-020-2665-2. 32805734 PMC7116492

[29] Pallesen J, Wang N, Corbett KS, Wrapp D, Kirchdoerfer RN, Turner HL, Cottrell CA, Becker MM, Wang L, Shi W, Kong W-P, Andres EL, Kettenbach AN, Denison MR, Chappell JD, Graham BS, Ward AB, McLellan JS. 2017. Immunogenicity and structures of a rationally designed prefusion MERS-CoV spike antigen. Proc Natl Acad Sci USA 114:E7348–E7357. doi:10.1073/pnas.1707304114. 28807998 PMC5584442

[30] Belouzard S, Millet JK, Licitra BN, Whittaker GR. 2012. Mechanisms of coronavirus cell entry mediated by the viral spike protein. Viruses 4:1011–1033. doi:10.3390/v4061011. 22816037 PMC3397359

[31] Jaimes JA, André NM, Chappie JS, Millet JK, Whittaker GR. 2020. Phylogenetic analysis and structural modeling of SARS-CoV-2 spike protein reveals an evolutionary distinct and proteolytically sensitive activation loop. J Mol Biol 432:3309–3325. doi:10.1016/j.jmb.2020.04.009. 32320687 PMC7166309

[32] Hsieh C-L, Goldsmith JA, Schaub JM, DiVenere AM, Kuo H-C, Javanmardi K, Le KC, Wrapp D, Lee AG, Liu Y, Chou C-W, Byrne PO, Hjorth CK, Johnson NV, Ludes-Meyers J, Nguyen AW, Park J, Wang N, Amengor D, Lavinder JJ, Ippolito GC, Maynard JA, Finkelstein IJ, McLellan JS. 2020. Structure-based design of prefusion-stabilized SARS-CoV-2 spikes. Science 369:1501–1505. doi:10.1126/science.abd0826. 32703906 PMC7402631

[33] Bos R, Rutten L, van der Lubbe JEM, Bakkers MJG, Hardenberg G, Wegmann F, Zuijdgeest D, de Wilde AH, Koornneef A, Verwilligen A, van Manen D, Kwaks T, Vogels R, Dalebout TJ, Myeni SK, Kikkert M, Snijder EJ, Li Z, Barouch DH, Vellinga J, Langedijk JPM, Zahn RC, Custers J, Schuitemaker H. 2020. Ad26 vector-based COVID-19 vaccine encoding a prefusion-stabilized SARS-CoV-2 spike immunogen induces potent humoral and cellular immune responses. NPJ Vaccines 5:91. doi:10.1038/s41541-020-00243-x. 33083026 PMC7522255

[34] Marrack P, McKee AS, Munks MW. 2009. Towards an understanding of the adjuvant action of aluminium. Nat Rev Immunol 9:287–293. doi:10.1038/nri2510. 19247370 PMC3147301

[35] Hayashi T, Momota M, Kuroda E, Kusakabe T, Kobari S, Makisaka K, Ohno Y, Suzuki Y, Nakagawa F, Lee MSJ, Coban C, Onodera R, Higashi T, Motoyama K, Ishii KJ, Arima H. 2018. DAMP-inducing adjuvant and PAMP adjuvants parallelly enhance protective type-2 and type-1 immune responses to influenza split vaccination. Front Immunol 9:2619. doi:10.3389/fimmu.2018.02619. 30515151 PMC6255964

[36] Wang Z-B, Xu J. 2020. Better adjuvants for better vaccines: progress in adjuvant delivery systems, modifications, and adjuvant–antigen codelivery. Vaccines (Basel) 8:128. doi:10.3390/vaccines8010128. 32183209 PMC7157724

[37] Rando HM, Greene CS, Robson MP, Boca SM, Wellhausen N, Lordan R, Brueffer C, Ray S, D’Agostino McGowan L, Gitter A, Dattoli AA, Velazquez R, Barton JP, Field JM, Ramsundar B, MacLean AL, Lee AJ, Immunology Institute of the Icahn School of Medicine, Hu F, Jadavji NM, Sell E, Wang J, Rafizadeh DN, Skelly AN, Ben Guebila M, Kolla L, Manheim D, Ghosh S, Byrd JB, Park Y, Bansal V, Capone S, Dziak JJ, Sun Y, Qi Y, Shinholster L, Lukan T, Knyazev S, Perrin D, Mangul S, Das S, Szeto GL, Lubiana T, Mai D, Goel RR, Boerckel JD, Naik A, Sun Y, Himmelstein DS, Kamil JP, Meyer JG, Mundo AI, COVID-19 Review Consortium. 2023. SARS-CoV-2 and COVID-19: an evolving review of diagnostics and therapeutics. Manubot. https://greenelab.github.io/covid19-review/.

[38] Hobernik D, Bros M. 2018. DNA vaccines—how far from clinical use? Int J Mol Sci 19:3605. doi:10.3390/ijms19113605. 30445702 PMC6274812

[39] Ghaffarifar F. 2018. Plasmid DNA vaccines: where are we now? Drugs Today (Barc) 54:315–333. doi:10.1358/dot.2018.54.5.2807864. 29911696

[40] Glenting J, Wessels S. 2005. Ensuring safety of DNA vaccines. Microb Cell Fact 4:26. doi:10.1186/1475-2859-4-26. 16144545 PMC1215512

[41] Williams J. 2013. Vector design for improved DNA vaccine efficacy, safety and production. Vaccines (Basel) 1:225–249. doi:10.3390/vaccines1030225. 26344110 PMC4494225

[42] Lim M, Badruddoza AZM, Firdous J, Azad M, Mannan A, Al-Hilal TA, Cho C-S, Islam MA. 2020. Engineered nanodelivery systems to improve DNA vaccine technologies. Pharmaceutics 12:30. doi:10.3390/pharmaceutics12010030. 31906277 PMC7022884

[43] World Health Organization. 2023. DNA vaccines. https://www.who.int/teams/health-product-policy-and-standards/standards-and-specifications/vaccines-quality/dna. Accessed 5 August 2022.

[44] Lapuente D, Stab V, Storcksdieck Genannt Bonsmann M, Maaske A, Köster M, Xiao H, Ehrhardt C, Tenbusch M. 2020. Innate signalling molecules as genetic adjuvants do not alter the efficacy of a DNA-based influenza A vaccine. PLoS One 15:e0231138. doi:10.1371/journal.pone.0231138. 32243477 PMC7122823

[45] Liu MA, Ulmer JB. 2005. Human clinical trials of plasmid DNA vaccines. Adv Genet 55:25–40. doi:10.1016/S0065-2660(05)55002-8. 16291211

[46] Roy MJ, Wu MS, Barr LJ, Fuller JT, Tussey LG, Speller S, Culp J, Burkholder JK, Swain WF, Dixon RM, Widera G, Vessey R, King A, Ogg G, Gallimore A, Haynes JR, Heydenburg FD. 2000. Induction of antigen-specific CD8+ T cells, T helper cells, and protective levels of antibody in humans by particle-mediated administration of a hepatitis B virus DNA vaccine. Vaccine 19:764–778. doi:10.1016/s0264-410x(00)00302-9. 11115698

[47] Weiner DB, Nabel GJ. 2018. Development of gene-based vectors for immunization, p 1305–1319.e8. *In* Plotkin SA, Orenstein WA, Offit PA, Edwards KM (ed), Plotkin’s vaccines, 7th ed. Elsevier, Philadelphia, PA.

[48] Gómez LA, Oñate AA. 2019. Plasmid-based DNA vaccines. *In* Gull M (ed), Plasmid. IntechOpen. doi:10.5772/intechopen.76754.

[49] Eusébio D, Neves AR, Costa D, Biswas S, Alves G, Cui Z, Sousa Â. 2021. Methods to improve the immunogenicity of plasmid DNA vaccines. Drug Discov Today 26:2575–2592. doi:10.1016/j.drudis.2021.06.008. 34214667

[50] Garver KA, LaPatra SE, Kurath G. 2005. Efficacy of an infectious hematopoietic necrosis (IHN) virus DNA vaccine in Chinook Oncorhynchus tshawytscha and sockeye O. nerka salmon. Dis Aquat Organ 64:13–22. doi:10.3354/dao064013. 15900683

[51] Thacker EL, Holtkamp DJ, Khan AS, Brown PA, Draghia-Akli R. 2006. Plasmid-mediated growth hormone-releasing hormone efficacy in reducing disease associated with Mycoplasma hyopneumoniae and porcine reproductive and respiratory syndrome virus infection. J Anim Sci 84:733–742. doi:10.2527/2006.843733x. 16478966

[52] Davidson AH, Traub-Dargatz JL, Rodeheaver RM, Ostlund EN, Pedersen DD, Moorhead RG, Stricklin JB, Dewell RD, Roach SD, Long RE, Albers SJ, Callan RJ, Salman MD. 2005. Immunologic responses to West Nile virus in vaccinated and clinically affected horses. J Am Vet Med Assoc 226:240–245. doi:10.2460/javma.2005.226.240. 15706975

[53] Langellotti CA, Gammella M, Soria I, Bellusci C, Quattrocchi V, Vermeulen M, Mongini C, Zamorano PI. 2021. An improved DNA vaccine against bovine herpesvirus-1 using CD40L and a chemical adjuvant induces specific cytotoxicity in mice. Viral Immunol 34:68–78. doi:10.1089/vim.2020.0082. 33146595

[54] Collins C, Lorenzen N, Collet B. 2019. DNA vaccination for finfish aquaculture. Fish Shellfish Immunol 85:106–125. doi:10.1016/j.fsi.2018.07.012. 30017931

[55] Chakraborty C, Agoramoorthy G. 2020. India’s cost-effective COVID-19 vaccine development initiatives. Vaccine 38:7883–7884. doi:10.1016/j.vaccine.2020.10.056. 33129610 PMC7574682

[56] Mallapaty S. 2021. India’s DNA COVID vaccine is a world first—more are coming. Nature 597:161–162. doi:10.1038/d41586-021-02385-x. 34475553

[57] Abbasi J. 2021. India’s new COVID-19 DNA vaccine for adolescents and adults is a first. JAMA 326:1365. doi:10.1001/jama.2021.16625. 34636874

[58] Inovio Pharmaceuticals. 2020. Phase 1 open-label study to evaluate the safety, tolerability and immunogenicity of INO-4800, a prophylactic vaccine against SARS-CoV-2, administered intradermally followed by electroporation in healthy volunteers. https://clinicaltrials.gov/ct2/show/NCT04336410. Accessed 8 February 2021.

[59] Diehl MC, Lee JC, Daniels SE, Tebas P, Khan AS, Giffear M, Sardesai NY, Bagarazzi ML. 2013. Tolerability of intramuscular and intradermal delivery by CELLECTRA adaptive constant current electroporation device in healthy volunteers. Hum Vaccin Immunother 9:2246–2252. doi:10.4161/hv.24702. 24051434 PMC3906411

[60] Sardesai NY, Weiner DB. 2011. Electroporation delivery of DNA vaccines: prospects for success. Curr Opin Immunol 23:421–429. doi:10.1016/j.coi.2011.03.008. 21530212 PMC3109217

[61] Tebas P, Yang S, Boyer JD, Reuschel EL, Patel A, Christensen-Quick A, Andrade VM, Morrow MP, Kraynyak K, Agnes J, Purwar M, Sylvester A, Pawlicki J, Gillespie E, Maricic I, Zaidi FI, Kim KY, Dia Y, Frase D, Pezzoli P, Schultheis K, Smith TRF, Ramos SJ, McMullan T, Buttigieg K, Carroll MW, Ervin J, Diehl MC, Blackwood E, Mammen MP, Lee J, Dallas MJ, Brown AS, Shea JE, Kim JJ, Weiner DB, Broderick KE, Humeau LM. 2021. Safety and immunogenicity of INO-4800 DNA vaccine against SARS-CoV-2: a preliminary report of an open-label, phase 1 clinical trial. EClinicalMedicine 31:100689. doi:10.1016/j.eclinm.2020.100689. 33392485 PMC7759123

[62] Mammen MP, Jr, Tebas P, Agnes J, Giffear M, Kraynyak KA, Blackwood E, Amante D, Reuschel EL, Purwar M, Christensen-Quick A, Liu N, Andrade VM, Carter J, Garufi G, Diehl MC, Sylvester A, Morrow MP, Pezzoli P, Kulkarni AJ, Zaidi FI, Frase D, Liaw K, Badie H, Simon KO, Smith TRF, Ramos S, Spitz R, Juba RJ, Lee J, Dallas M, Brown AS, Shea JE, Kim JJ, Weiner DB, Broderick KE, Boyer JD, Humeau LM. 7 May 2021. Safety and immunogenicity of INO-4800 DNA vaccine against SARS-CoV-2: a preliminary report of a randomized, blinded, placebo-controlled, phase 2 clinical trial in adults at high risk of viral exposure. medRxiv. doi:10.1101/2021.05.07.21256652.

[63] Inovio. 9 November 2021. INOVIO receives U.S. FDA authorization to proceed with INNOVATE phase 3 segment for its COVID-19 vaccine candidate, INO-4800, in the U.S. https://ir.inovio.com/news-releases/news-releases-details/2021/INOVIO-Receives-U.S.-FDA-Authorization-to-Proceed-with-INNOVATE-Phase-3-Segment-for-its-COVID-19-Vaccine-Candidate-INO-4800-in-the-U.S/default.aspx. Accessed 10 October 2022.

[64] Inovio. 3 November 2021. INOVIO further expands INNOVATE phase 3 trial for COVID-19 DNA vaccine candidate INO-4800 with regulatory authorization from India. https://ir.inovio.com/news-releases/news-releases-details/2021/INOVIO-Further-Expands-INNOVATE-Phase-3-Trial-for-COVID-19-DNA-Vaccine-Candidate-INO-4800-With-Regulatory-Authorization-from-India/default.aspx. Accessed 10 October 2022.

[65] Inovio. 11 October 2021. INOVIO expands INNOVATE phase 3 for INO-4800, its DNA vaccine candidate for COVID-19, to include Colombia following regulatory authorization. https://ir.inovio.com/news-releases/news-releases-details/2021/INOVIO-Expands-INNOVATE-Phase-3-for-INO-4800-its-DNA-Vaccine-Candidate-for-COVID-19-to-include-Colombia-following-Regulatory-Authorization/default.aspx. Accessed 10 October 2022.

[66] Inovio. 22 September 2021. INOVIO receives regulatory authorization to conduct phase 3 efficacy trial of its COVID-19 DNA vaccine candidate, INO-4800, in Mexico. https://ir.inovio.com/news-releases/news-releases-details/2021/INOVIO-Receives-Regulatory-Authorization-to-Conduct-Phase-3-Efficacy-Trial-of-its-COVID-19-DNA-Vaccine-Candidate-INO-4800-in-Mexico/default.aspx. Accessed 10 October 2022.

[67] Momin T, Kansagra K, Patel H, Sharma S, Sharma B, Patel J, Mittal R, Sanmukhani J, Maithal K, Dey A, Chandra H, Rajanathan CT, Pericherla HP, Kumar P, Narkhede A, Parmar D. 2021. Safety and immunogenicity of a DNA SARS-CoV-2 vaccine (ZyCoV-D): results of an open-label, non-randomized phase I part of phase I/II clinical study by intradermal route in healthy subjects in India. EClinicalMedicine 38:101020. doi:10.1016/j.eclinm.2021.101020. 34308319 PMC8285262

[68] Khobragade A, Bhate S, Ramaiah V, Deshpande S, Giri K, Phophle H, Supe P, Godara I, Revanna R, Nagarkar R, Sanmukhani J, Dey A, Rajanathan TMC, Kansagra K, Koradia P, ZyCoV-D phase 3 Study Investigator Group. 2022. Efficacy, safety, and immunogenicity of the DNA SARS-CoV-2 vaccine (ZyCoV-D): the interim efficacy results of a phase 3, randomised, double-blind, placebo-controlled study in India. Lancet 399:1313–1321. doi:10.1016/S0140-6736(22)00151-9. 35367003 PMC8970574

[69] VIPER Group COVID19 Vaccine Tracker Team. 2022. COVID19 Vaccine Tracker. Zydus Cadila: ZyCoV-D. https://covid19.trackvaccines.org/vaccines/29/. Accessed 10 October 2022.

[70] Mathieu E, Ritchie H, Ortiz-Ospina E, Roser M, Hasell J, Appel C, Giattino C, Rodés-Guirao L. 2021. A global database of COVID-19 vaccinations. Nat Hum Behav 5:947–953. doi:10.1038/s41562-021-01122-8. 33972767

[71] Chandna H. 3 December 2021. Covishield, Covaxin, ZyCoV-D makers to assess efficacy of vaccine as they await data on Omicron. News18, Uttar Pradesh, India. https://www.news18.com/news/india/covishield-covaxin-zycov-d-makers-to-assess-efficacy-of-their-vaccine-as-they-await-data-on-omicron-4504295.html. Accessed 10 October 2022.

[72] Andrade VM, Christensen-Quick A, Agnes J, Tur J, Reed C, Kalia R, Marrero I, Elwood D, Schultheis K, Purwar M, Reuschel E, McMullan T, Pezzoli P, Kraynyak K, Sylvester A, Mammen MP, Tebas P, Joseph Kim J, Weiner DB, Smith TRF, Ramos SJ, Humeau LM, Boyer JD, Broderick KE. 2021. INO-4800 DNA vaccine induces neutralizing antibodies and T cell activity against global SARS-CoV-2 variants. NPJ Vaccines 6:121. doi:10.1038/s41541-021-00384-7. 34650089 PMC8516974

[73] Kraynyak KA, Blackwood E, Agnes J, Tebas P, Giffear M, Amante D, Reuschel EL, Purwar M, Christensen-Quick A, Liu N, Andrade VM, Diehl MC, Wani S, Lupicka M, Sylvester A, Morrow MP, Pezzoli P, McMullan T, Kulkarni AJ, Zaidi FI, Frase D, Liaw K, Smith TRF, Ramos SJ, Ervin J, Adams M, Lee J, Dallas M, Shah Brown A, Shea JE, Kim JJ, Weiner DB, Broderick KE, Humeau LM, Boyer JD, Mammen MP, Jr. 2022. SARS-CoV-2 DNA vaccine INO-4800 induces durable immune responses capable of being boosted in a phase 1 open-label trial. J Infect Dis 225:1923–1932. doi:10.1093/infdis/jiac016. 35079784 PMC8807286

[74] Inovio. 30 November 2021. INOVIO announces strategy to address Omicron (B.1.1.529) and future SARS-CoV-2 variants. https://www.prnewswire.com/news-releases/inovio-announces-strategy-to-address-omicron-b1-1-529-and-future-sars-cov-2-variants-301433776.html. Accessed 10 October 2022.

[75] Walters JN, Schouest B, Patel A, Reuschel EL, Schultheis K, Parzych E, Maricic I, Gary EN, Purwar M, Andrade VM, Doan A, Elwood D, Eblimit Z, Nguyen B, Frase D, Zaidi FI, Kulkarni A, Generotti A, Joseph Kim J, Humeau LM, Ramos SJ, Smith TRF, Weiner DB, Broderick KE. 2022. Prime-boost vaccination regimens with INO-4800 and INO-4802 augment and broaden immune responses against SARS-CoV-2 in nonhuman primates. Vaccine 40:2960–2969. doi:10.1016/j.vaccine.2022.03.060. 35428500 PMC8977452

[76] Reed CC, Schultheis K, Andrade VM, Kalia R, Tur J, Schouest B, Elwood D, Walters JN, Maricic I, Doan A, Vazquez M, Eblimit Z, Pezzoli P, Amante D, Porto M, Narvaez B, Lok M, Spence B, Bradette H, Horn H, Yang M, Fader J, Ferrer R, Weiner DB, Kar S, Kim JJ, Humeau LM, Ramos SJ, Smith TRF, Broderick KE. 4 August 2021. Design, immunogenicity and efficacy of a pan-SARS-CoV-2 synthetic DNA vaccine. bioRxiv. doi:10.1101/2021.05.11.443592.

[77] Lauer KB, Borrow R, Blanchard TJ. 2017. Multivalent and multipathogen viral vector vaccines. Clin Vaccine Immunol 24:e00298-16. doi:10.1128/CVI.00298-16. 27535837 PMC5216423

[78] Ewer KJ, Lambe T, Rollier CS, Spencer AJ, Hill AV, Dorrell L. 2016. Viral vectors as vaccine platforms: from immunogenicity to impact. Curr Opin Immunol 41:47–54. doi:10.1016/j.coi.2016.05.014. 27286566

[79] Antrobus RD, Coughlan L, Berthoud TK, Dicks MD, Hill AV, Lambe T, Gilbert SC. 2014. Clinical assessment of a novel recombinant simian adenovirus ChAdOx1 as a vectored vaccine expressing conserved influenza A antigens. Mol Ther 22:668–674. doi:10.1038/mt.2013.284. 24374965 PMC3944330

[80] Al-Kassmy J, Pedersen J, Kobinger G. 2020. Vaccine candidates against coronavirus infections. Where does COVID-19 stand? Viruses 12:861. doi:10.3390/v12080861. 32784685 PMC7472384

[81] Pastoret P-P, Vanderplasschen A. 2003. Poxviruses as vaccine vectors. Comp Immunol Microbiol Infect Dis 26:343–355. doi:10.1016/S0147-9571(03)00019-5. 12818621

[82] García-Arriaza J, Esteban M. 2014. Enhancing poxvirus vectors vaccine immunogenicity. Hum Vaccin Immunother 10:2235–2244. doi:10.4161/hv.28974. 25424927 PMC4896794

[83] Lasaro MO, Ertl HC. 2009. New insights on adenovirus as vaccine vectors. Mol Ther 17:1333–1339. doi:10.1038/mt.2009.130. 19513019 PMC2835230

[84] Roberts A, Buonocore L, Price R, Forman J, Rose JK. 1999. Attenuated vesicular stomatitis viruses as vaccine vectors. J Virol 73:3723–3732. doi:10.1128/JVI.73.5.3723-3732.1999. 10196265 PMC104148

[85] Lichty BD, Power AT, Stojdl DF, Bell JC. 2004. Vesicular stomatitis virus: re-inventing the bullet. Trends Mol Med 10:210–216. doi:10.1016/j.molmed.2004.03.003. 15121047

[86] Rollier CS, Reyes-Sandoval A, Cottingham MG, Ewer K, Hill AV. 2011. Viral vectors as vaccine platforms: deployment in sight. Curr Opin Immunol 23:377–382. doi:10.1016/j.coi.2011.03.006. 21514130

[87] Nayak S, Herzog RW. 2010. Progress and prospects: immune responses to viral vectors. Gene Ther 17:295–304. doi:10.1038/gt.2009.148. 19907498 PMC3044498

[88] Ura T, Okuda K, Shimada M. 2014. Developments in viral vector-based vaccines. Vaccines (Basel) 2:624–641. doi:10.3390/vaccines2030624. 26344749 PMC4494222

[89] Moffatt S, Hays J, HogenEsch H, Mittal SK. 2000. Circumvention of vector-specific neutralizing antibody response by alternating use of human and non-human adenoviruses: implications in gene therapy. Virology 272:159–167. doi:10.1006/viro.2000.0350. 10873758

[90] Fausther-Bovendo H, Kobinger GP. 2014. Pre-existing immunity against Ad vectors: humoral, cellular, and innate response, what’s important? Hum Vaccin Immunother 10:2875–2884. doi:10.4161/hv.29594. 25483662 PMC5443060

[91] Vrba SM, Kirk NM, Brisse ME, Liang Y, Ly H. 2020. Development and applications of viral vectored vaccines to combat zoonotic and emerging public health threats. Vaccines (Basel) 8:680. doi:10.3390/vaccines8040680. 33202961 PMC7712223

[92] Ollmann Saphire E. 2020. A vaccine against Ebola virus. Cell 181:6. doi:10.1016/j.cell.2020.03.011. 32243796

[93] Bliss CM, Drammeh A, Bowyer G, Sanou GS, Jagne YJ, Ouedraogo O, Edwards NJ, Tarama C, Ouedraogo N, Ouedraogo M, Njie-Jobe J, Diarra A, Afolabi MO, Tiono AB, Yaro JB, Adetifa UJ, Hodgson SH, Anagnostou NA, Roberts R, Duncan CJA, Cortese R, Viebig NK, Leroy O, Lawrie AM, Flanagan KL, Kampmann B, Imoukhuede EB, Sirima SB, Bojang K, Hill AVS, Nébié I, Ewer KJ. 2017. Viral vector malaria vaccines induce high-level T cell and antibody responses in West African children and infants. Mol Ther 25:547–559. doi:10.1016/j.ymthe.2016.11.003. 28153101 PMC5368405

[94] Li S, Locke E, Bruder J, Clarke D, Doolan DL, Havenga MJE, Hill AVS, Liljestrom P, Monath TP, Naim HY, Ockenhouse C, Tang DC, Van Kampen KR, Viret J-F, Zavala F, Dubovsky F. 2007. Viral vectors for malaria vaccine development. Vaccine 25:2567–2574. doi:10.1016/j.vaccine.2006.07.035. 16914237 PMC7131149

[95] Ledgerwood JE, DeZure AD, Stanley DA, Coates EE, Novik L, Enama ME, Berkowitz NM, Hu Z, Joshi G, Ploquin A, Sitar S, Gordon IJ, Plummer SA, Holman LA, Hendel CS, Yamshchikov G, Roman F, Nicosia A, Colloca S, Cortese R, Bailer RT, Schwartz RM, Roederer M, Mascola JR, Koup RA, Sullivan NJ, Graham BS, VRC 207 Study Team. 2017. Chimpanzee adenovirus vector Ebola vaccine. N Engl J Med 376:928–938. doi:10.1056/NEJMoa1410863. 25426834

[96] Geisbert TW, Feldmann H. 2011. Recombinant vesicular stomatitis virus–based vaccines against Ebola and Marburg virus infections. J Infect Dis 204:S1075–S1081. doi:10.1093/infdis/jir349. 21987744 PMC3218670

[97] Marzi A, Feldmann H. 2014. Ebola virus vaccines: an overview of current approaches. Expert Rev Vaccines 13:521–531. doi:10.1586/14760584.2014.885841. 24575870 PMC4785864

[98] Parks CL, Picker LJ, King CR. 2013. Development of replication-competent viral vectors for HIV vaccine delivery. Curr Opin HIV AIDS 8:402–411. doi:10.1097/COH.0b013e328363d389. 23925000 PMC4040527

[99] Trivedi S, Jackson RJ, Ranasinghe C. 2014. Different HIV pox viral vector-based vaccines and adjuvants can induce unique antigen presenting cells that modulate CD8 T cell avidity. Virology 468–470:479–489. doi:10.1016/j.virol.2014.09.004. 25261870

[100] See RH, Zakhartchouk AN, Petric M, Lawrence DJ, Mok CPY, Hogan RJ, Rowe T, Zitzow LA, Karunakaran KP, Hitt MM, Graham FL, Prevec L, Mahony JB, Sharon C, Auperin TC, Rini JM, Tingle AJ, Scheifele DW, Skowronski DM, Patrick DM, Voss TG, Babiuk LA, Gauldie J, Roper RL, Brunham RC, Finlay BB. 2006. Comparative evaluation of two severe acute respiratory syndrome (SARS) vaccine candidates in mice challenged with SARS coronavirus. J Gen Virol 87:641–650. doi:10.1099/vir.0.81579-0. 16476986

[101] See RH, Petric M, Lawrence DJ, Mok CPY, Rowe T, Zitzow LA, Karunakaran KP, Voss TG, Brunham RC, Gauldie J, Finlay BB, Roper RL. 2008. Severe acute respiratory syndrome vaccine efficacy in ferrets: whole killed virus and adenovirus-vectored vaccines. J Gen Virol 89:2136–2146. doi:10.1099/vir.0.2008/001891-0. 18753223

[102] Yeung HT. 2018. Update with the development of Ebola vaccines and implications of emerging evidence to inform future policy recommendations. World Health Organization. https://cdn.who.int/media/docs/default-source/biologicals/vaccine-standardization/ebola/1-ebola-vaccine-background-document.pdf.

[103] Alharbi NK, Padron-Regalado E, Thompson CP, Kupke A, Wells D, Sloan MA, Grehan K, Temperton N, Lambe T, Warimwe G, Becker S, Hill AVS, Gilbert SC. 2017. ChAdOx1 and MVA based vaccine candidates against MERS-CoV elicit neutralising antibodies and cellular immune responses in mice. Vaccine 35:3780–3788. doi:10.1016/j.vaccine.2017.05.032. 28579232 PMC5516308

[104] van Doremalen N, Haddock E, Feldmann F, Meade-White K, Bushmaker T, Fischer RJ, Okumura A, Hanley PW, Saturday G, Edwards NJ, Clark MHA, Lambe T, Gilbert SC, Munster VJ. 2020. A single dose of ChAdOx1 MERS provides protective immunity in rhesus macaques. Sci Adv 6:eaba8399. doi:10.1126/sciadv.aba8399. 32577525 PMC7286676

[105] Folegatti PM, Bittaye M, Flaxman A, Lopez FR, Bellamy D, Kupke A, Mair C, Makinson R, Sheridan J, Rohde C, Halwe S, Jeong Y, Park Y-S, Kim J-O, Song M, Boyd A, Tran N, Silman D, Poulton I, Datoo M, Marshall J, Themistocleous Y, Lawrie A, Roberts R, Berrie E, Becker S, Lambe T, Hill A, Ewer K, Gilbert S. 2020. Safety and immunogenicity of a candidate Middle East respiratory syndrome coronavirus viral-vectored vaccine: a dose-escalation, open-label, non-randomised, uncontrolled, phase 1 trial. Lancet Infect Dis 20:816–826. doi:10.1016/S1473-3099(20)30160-2. 32325038 PMC7172901

[106] van Doremalen N, Lambe T, Spencer A, Belij-Rammerstorfer S, Purushotham JN, Port JR, Avanzato VA, Bushmaker T, Flaxman A, Ulaszewska M, Feldmann F, Allen ER, Sharpe H, Schulz J, Holbrook M, Okumura A, Meade-White K, Pérez-Pérez L, Edwards NJ, Wright D, Bissett C, Gilbride C, Williamson BN, Rosenke R, Long D, Ishwarbhai A, Kailath R, Rose L, Morris S, Powers C, Lovaglio J, Hanley PW, Scott D, Saturday G, de Wit E, Gilbert SC, Munster VJ. 2020. ChAdOx1 nCoV-19 vaccine prevents SARS-CoV-2 pneumonia in rhesus macaques. Nature 586:578–582. doi:10.1038/s41586-020-2608-y. 32731258 PMC8436420

[107] Folegatti PM, Ewer KJ, Aley PK, Angus B, Becker S, Belij-Rammerstorfer S, Bellamy D, Bibi S, Bittaye M, Clutterbuck EA, Dold C, Faust SN, Finn A, Flaxman AL, Hallis B, Heath P, Jenkin D, Lazarus R, Makinson R, Minassian AM, Pollock KM, Ramasamy M, Robinson H, Snape M, Tarrant R, Voysey M, Green C, Douglas AD, Hill AVS, Lambe T, Gilbert SC, Pollard AJ, Oxford COVID Vaccine Trial Group. 2020. Safety and immunogenicity of the ChAdOx1 nCoV-19 vaccine against SARS-CoV-2: a preliminary report of a phase 1/2, single-blind, randomised controlled trial. Lancet 396:467–478. doi:10.1016/S0140-6736(20)31604-4. 32702298 PMC7445431

[108] Jones I, Roy P. 2021. Sputnik V COVID-19 vaccine candidate appears safe and effective. Lancet 397:642–643. doi:10.1016/S0140-6736(21)00191-4. 33545098 PMC7906719

[109] Barouch DH, Kik SV, Weverling GJ, Dilan R, King SL, Maxfield LF, Clark S, Ng’ang’a D, Brandariz KL, Abbink P, Sinangil F, de Bruyn G, Gray GE, Roux S, Bekker L-G, Dilraj A, Kibuuka H, Robb ML, Michael NL, Anzala O, Amornkul PN, Gilmour J, Hural J, Buchbinder SP, Seaman MS, Dolin R, Baden LR, Carville A, Mansfield KG, Pau MG, Goudsmit J. 2011. International seroepidemiology of adenovirus serotypes 5, 26, 35, and 48 in pediatric and adult populations. Vaccine 29:5203–5209. doi:10.1016/j.vaccine.2011.05.025. 21619905 PMC3138857

[110] US Government Accountability Office. 11 February 2021. Operation Warp Speed: accelerated COVID-19 vaccine development status and efforts to address manufacturing challenges. https://www.gao.gov/products/gao-21-319. Accessed 10 October 2022.

[111] Johnson & Johnson. 30 March 2020. Johnson & Johnson announces a lead vaccine candidate for COVID-19; landmark new partnership with U.S. Department of Health & Human Services; and commitment to supply one billion vaccines worldwide for emergency pandemic use. https://www.jnj.com/johnson-johnson-announces-a-lead-vaccine-candidate-for-covid-19-landmark-new-partnership-with-u-s-department-of-health-human-services-and-commitment-to-supply-one-billion-vaccines-worldwide-for-emergency-pandemic-use. Accessed 10 October 2022.

[112] Sadoff J, Le Gars M, Shukarev G, Heerwegh D, Truyers C, de Groot AM, Stoop J, Tete S, Van Damme W, Leroux-Roels I, Berghmans P-J, Kimmel M, Van Damme P, de Hoon J, Smith W, Stephenson KE, De Rosa SC, Cohen KW, McElrath MJ, Cormier E, Scheper G, Barouch DH, Hendriks J, Struyf F, Douoguih M, Van Hoof J, Schuitemaker H. 2021. Interim results of a phase 1–2a trial of Ad26.COV2.S Covid-19 vaccine. N Engl J Med 384:1824–1835. doi:10.1056/NEJMoa2034201. 33440088 PMC7821985

[113] Mercado NB, Zahn R, Wegmann F, Loos C, Chandrashekar A, Yu J, Liu J, Peter L, McMahan K, Tostanoski LH, He X, Martinez DR, Rutten L, Bos R, van Manen D, Vellinga J, Custers J, Langedijk JP, Kwaks T, Bakkers MJG, Zuijdgeest D, Rosendahl Huber SK, Atyeo C, Fischinger S, Burke JS, Feldman J, Hauser BM, Caradonna TM, Bondzie EA, Dagotto G, Gebre MS, Hoffman E, Jacob-Dolan C, Kirilova M, Li Z, Lin Z, Mahrokhian SH, Maxfield LF, Nampanya F, Nityanandam R, Nkolola JP, Patel S, Ventura JD, Verrington K, Wan H, Pessaint L, Van Ry A, Blade K, Strasbaugh A, Cabus M, Brown R, Cook A, Zouantchangadou S, Teow E, Andersen H, Lewis MG, Cai Y, Chen B, Schmidt AG, Reeves RK, Baric RS, Lauffenburger DA, Alter G, Stoffels P, Mammen M, Van Hoof J, Schuitemaker H, Barouch DH. 2020. Single-shot Ad26 vaccine protects against SARS-CoV-2 in rhesus macaques. Nature 586:583–588. doi:10.1038/s41586-020-2607-z. 32731257 PMC7581548

[114] Tostanoski LH, Wegmann F, Martinot AJ, Loos C, McMahan K, Mercado NB, Yu J, Chan CN, Bondoc S, Starke CE, Nekorchuk M, Busman-Sahay K, Piedra-Mora C, Wrijil LM, Ducat S, Custers J, Atyeo C, Fischinger S, Burke JS, Feldman J, Hauser BM, Caradonna TM, Bondzie EA, Dagotto G, Gebre MS, Jacob-Dolan C, Lin Z, Mahrokhian SH, Nampanya F, Nityanandam R, Pessaint L, Porto M, Ali V, Benetiene D, Tevi K, Andersen H, Lewis MG, Schmidt AG, Lauffenburger DA, Alter G, Estes JD, Schuitemaker H, Zahn R, Barouch DH. 2020. Ad26 vaccine protects against SARS-CoV-2 severe clinical disease in hamsters. Nat Med 26:1694–1700. doi:10.1038/s41591-020-1070-6. 32884153 PMC7671939

[115] Solforosi L, Kuipers H, Huber SKR, van der Lubbe JEM, Dekking L, Czapska-Casey DN, Gil AI, Baert MRM, Drijver J, Vaneman J, van Huizen E, Choi Y, Vreugdenhil J, Kroos S, de Wilde AH, Kourkouta E, Custers J, Dalebout TJ, Myeni SK, Kikkert M, Snijder EJ, Barouch DH, Böszörményi KP, Stammes MA, Kondova I, Verschoor EJ, Verstrepen BE, Koopman G, Mooij P, Bogers WMJM, van Heerden M, Muchene L, Tolboom JTBM, Roozendaal R, Schuitemaker H, Wegmann F, Zahn RC. 17 November 2020. Immunogenicity and protective efficacy of one- and two-dose regimens of the Ad26.COV2.S COVID-19 vaccine candidate in adult and aged rhesus macaques. bioRxiv. doi:10.1101/2020.11.17.368258.

[116] Roozendaal R, Solforosi L, Stieh D, Serroyen J, Straetemans R, Wegmann F, Rosendahl Huber SK, van der Lubbe JEM, Hendriks J, Le Gars M, Dekking L, Czapska-Casey DN, Guimera N, Janssen S, Tete S, Chandrashekar A, Mercado N, Yu J, Koudstaal W, Sadoff J, Barouch DH, Schuitemaker H, Zahn R. 30 January 2021. SARS-CoV-2 binding and neutralizing antibody levels after vaccination with Ad26.COV2.S predict durable protection in rhesus macaques. bioRxiv. doi:10.1101/2021.01.30.428921. PMC8497464 34620860

[117] Sadoff J, Gray G, Vandebosch A, Cárdenas V, Shukarev G, Grinsztejn B, Goepfert PA, Truyers C, Van Dromme I, Spiessens B, Vingerhoets J, Custers J, Scheper G, Robb ML, Treanor J, Ryser MF, Barouch DH, Swann E, Marovich MA, Neuzil KM, Corey L, Stoddard J, Hardt K, Ruiz-Guiñazú J, Le Gars M, Schuitemaker H, Van Hoof J, Struyf F, Douoguih M, ENSEMBLE Study Group. 2022. Final analysis of efficacy and safety of single-dose Ad26.COV2.S. N Engl J Med 386:847–860. doi:10.1056/NEJMoa2117608. 35139271 PMC8849184

[118] AstraZeneca. 30 December 2020. AstraZeneca’s COVID-19 vaccine authorised for emergency supply in the UK. https://www.astrazeneca.com/media-centre/press-releases/2020/astrazenecas-covid-19-vaccine-authorised-in-uk.html. Accessed 10 October 2022.

[119] The Brussels Times. 11 January 2021. 1.5 million people have received Sputnik V vaccine, Russia says. The Brussels Times, Brussels, Belgium. https://www.brusselstimes.com/news-contents/world/149039/1-5-million-people-have-received-sputnik-v-vaccine-russia-says-russian-direct-investment-fund-mikhail-murashko. Accessed 10 October 2022.

[120] Daventry M. 2 December 2021. Hungary becomes first EU country to deploy Russia’s COVID-19 vaccine. euronews, Lyon, France. https://www.euronews.com/2021/02/12/hungary-to-begin-using-russia-s-sputnik-v-vaccine-today. Accessed 10 October 2022.

[121] AP. 24 February 2021. San Marino buys Russia’s Sputnik V after EU vaccine delivery delays. euronews, Lyon, France. https://www.euronews.com/2021/02/24/san-marino-buys-russia-s-sputnik-v-after-eu-vaccine-delivery-delays. Accessed 10 October 2022.

[122] AFP. 29 December 2020. Belarus starts coronavirus vaccination with Sputnik V. The Moscow Times. https://www.themoscowtimes.com/2020/12/29/belarus-starts-coronavirus-vaccination-with-sputnik-v-a72512. Accessed 10 October 2022.

[123] Logunov DY, Dolzhikova IV, Shcheblyakov DV, Tukhvatulin AI, Zubkova OV, Dzharullaeva AS, Kovyrshina AV, Lubenets NL, Grousova DM, Erokhova AS, Botikov AG, Izhaeva FM, Popova O, Ozharovskaya TA, Esmagambetov IB, Favorskaya IA, Zrelkin DI, Voronina DV, Shcherbinin DN, Semikhin AS, Simakova YV, Tokarskaya EA, Egorova DA, Shmarov MM, Nikitenko NA, Gushchin VA, Smolyarchuk EA, Zyryanov SK, Borisevich SV, Naroditsky BS, Gintsburg AL. 2021. Safety and efficacy of an rAd26 and rAd5 vector-based heterologous prime-boost COVID-19 vaccine: an interim analysis of a randomised controlled phase 3 trial in Russia. Lancet 397:671–681. doi:10.1016/S0140-6736(21)00234-8. 33545094 PMC7852454

[124] Sadoff J, Gray G, Vandebosch A, Cárdenas V, Shukarev G, Grinsztejn B, Goepfert PA, Truyers C, Fennema H, Spiessens B, Offergeld K, Scheper G, Taylor KL, Robb ML, Treanor J, Barouch DH, Stoddard J, Ryser MF, Marovich MA, Neuzil KM, Corey L, Cauwenberghs N, Tanner T, Hardt K, Ruiz-Guiñazú J, Le Gars M, Schuitemaker H, Van Hoof J, Struyf F, Douoguih M, ENSEMBLE Study Group. 2021. Safety and efficacy of single-dose Ad26.COV2.S vaccine against Covid-19. N Engl J Med 384:2187–2201. doi:10.1056/NEJMoa2101544. 33882225 PMC8220996

[125] National Institutes of Health. 29 January 2021. Janssen investigational COVID-19 vaccine: interim analysis of phase 3 clinical data released. National Institutes of Health, Bethesda, MD. https://www.nih.gov/news-events/news-releases/janssen-investigational-covid-19-vaccine-interim-analysis-phase-3-clinical-data-released. Accessed 10 October 2022.

[126] Johnson & Johnson. 29 January 2021. Johnson & Johnson announces single-shot Janssen COVID-19 vaccine candidate met primary endpoints in interim analysis of its phase 3 ENSEMBLE trial. https://www.janssen.com/emea/sites/www_janssen_com_emea/files/johnson_johnson_announces_single-shot_janssen_covid-19_vaccine_candidate_met_primary_endpoints_in_interim_analysis_of_its_phase_3_ensemble_trial.pdf.

[127] Burki TK. 2020. The Russian vaccine for COVID-19. Lancet Respir Med 8:e85–e86. doi:10.1016/S2213-2600(20)30402-1. 32896274 PMC7837053

[128] Cohen J. 11 November 2020. Russia’s claim of a successful COVID-19 vaccine doesn’t pass the ‘smell test,’ critics say. American Association for the Advancement of Science (AAAS), Washington, DC. https://www.science.org/content/article/russia-s-claim-successful-covid-19-vaccine-doesn-t-pass-smell-test-critics-say.

[129] Callaway E. 11 November 2020. Russia announces positive COVID-vaccine results from controversial trial. Nature doi:10.1038/d41586-020-03209-0. 33177689

[130] Thorp HH. 2020. A dangerous rush for vaccines. Science 369:885. doi:10.1126/science.abe3147. 32792466

[131] Lovelace B, Jr. 11 August 2020. Scientists worry whether Russia’s ‘Sputnik V’ coronavirus vaccine is safe and effective. CNBC. https://www.cnbc.com/2020/08/11/scientists-worry-whether-russias-sputnik-v-coronavirus-vaccine-is-safe-and-effective.html. Accessed 10 October 2022.

[132] Logunov DY, Dolzhikova IV, Zubkova OV, Tukhvatulin AI, Shcheblyakov DV, Dzharullaeva AS, Grousova DM, Erokhova AS, Kovyrshina AV, Botikov AG, Izhaeva FM, Popova O, Ozharovskaya TA, Esmagambetov IB, Favorskaya IA, Zrelkin DI, Voronina DV, Shcherbinin DN, Semikhin AS, Simakova YV, Tokarskaya EA, Lubenets NL, Egorova DA, Shmarov MM, Nikitenko NA, Morozova LF, Smolyarchuk EA, Kryukov EV, Babira VF, Borisevich SV, Naroditsky BS, Gintsburg AL. 2020. Safety and immunogenicity of an rAd26 and rAd5 vector-based heterologous prime-boost COVID-19 vaccine in two formulations: two open, non-randomised phase 1/2 studies from Russia. Lancet 396:887–897. doi:10.1016/S0140-6736(20)31866-3. 32896291 PMC7471804

[133] Gamaleya Center, Russian Direct Investment Fund. Sputnik V. Clinical trials. https://sputnikvaccine.com/about-vaccine/clinical-trials/. Accessed 10 October 2022.

[134] Phillips N, Cyranoski D, Mallapaty S. 9 September 2020. A leading coronavirus vaccine trial is on hold: scientists react. Nature doi:10.1038/d41586-020-02594-w. 32908295

[135] Cyranoski D, Mallapaty S. 2020. Scientists relieved as coronavirus vaccine trial restarts—but question lack of transparency. Nature 585:331–332. doi:10.1038/d41586-020-02633-6. 32929259

[136] Robbins R, LaFraniere S, Weiland N, Kirkpatrick DD, Mueller B. 8 December 2020. Blunders eroded U.S. confidence in early vaccine front-runner. The New York Times, New York, NY. https://www.nytimes.com/2020/12/08/business/covid-vaccine-oxford-astrazeneca.html.

[137] Roberts M. 27 November 2020. Oxford/AstraZeneca Covid vaccine ‘dose error’ explained. BBC News. https://www.bbc.com/news/health-55086927.

[138] Sanchez S, Palacio N, Dangi T, Ciucci T, Penaloza-MacMaster P. 2021. Fractionating a COVID-19 Ad5-vectored vaccine improves virus-specific immunity. Sci Immunol 6:eabi8635. doi:10.1126/sciimmunol.abi8635. 34648369 PMC9278052

[139] Wolf ME, Luz B, Niehaus L, Bhogal P, Bäzner H, Henkes H. 2021. Thrombocytopenia and intracranial venous sinus thrombosis after “COVID-19 Vaccine AstraZeneca” exposure. J Clin Med 10:1599. doi:10.3390/jcm10081599. 33918932 PMC8069989

[140] Wise J. 2021. Covid-19: European countries suspend use of Oxford-AstraZeneca vaccine after reports of blood clots. BMJ 372:n699. doi:10.1136/bmj.n699. 33707182

[141] Mahase E. 2021. Covid-19: AstraZeneca vaccine is not linked to increased risk of blood clots, finds European Medicine Agency. BMJ 372:n774. doi:10.1136/bmj.n774. 33741638

[142] Mahase E. 2021. Covid-19: US suspends Johnson and Johnson vaccine rollout over blood clots. BMJ 373:n970. doi:10.1136/bmj.n970. 33849896

[143] Tanne JH. 2021. Covid-19: US authorises Johnson and Johnson vaccine again, ending pause in rollout. BMJ 373:n1079. doi:10.1136/bmj.n1079. 33903130

[144] Mahase E. 2021. Covid-19: unusual blood clots are “very rare side effect” of Janssen vaccine, says EMA. BMJ 373:n1046. doi:10.1136/bmj.n1046. 33883164

[145] Oliver SE, Wallace M, See I, Mbaeyi S, Godfrey M, Hadler SC, Jatlaoui TC, Twentyman E, Hughes MM, Rao AK, Fiore A, Su JR, Broder KR, Shimabukuro T, Lale A, Shay DK, Markowitz LE, Wharton M, Bell BP, Brooks O, McNally V, Lee GM, Talbot HK, Daley MF. 2022. Use of the Janssen (Johnson & Johnson) COVID-19 vaccine: updated interim recommendations from the Advisory Committee on Immunization Practices—United States, December 2021. MMWR Morb Mortal Wkly Rep 71:90–95. doi:10.15585/mmwr.mm7103a4. 35051137 PMC8774160

[146] Shay DK, Gee J, Su JR, Myers TR, Marquez P, Liu R, Zhang B, Licata C, Clark TA, Shimabukuro TT. 2021. Safety monitoring of the Janssen (Johnson & Johnson) COVID-19 vaccine—United States, March–April 2021. MMWR Morb Mortal Wkly Rep 70:680–684. doi:10.15585/mmwr.mm7018e2. 33956784 PMC9368748

[147] Rosenblum HG, Hadler SC, Moulia D, Shimabukuro TT, Su JR, Tepper NK, Ess KC, Woo EJ, Mba-Jonas A, Alimchandani M, Nair N, Klein NP, Hanson KE, Markowitz LE, Wharton M, McNally VV, Romero JR, Talbot HK, Lee GM, Daley MF, Mbaeyi SA, Oliver SE. 2021. Use of COVID-19 vaccines after reports of adverse events among adult recipients of Janssen (Johnson & Johnson) and mRNA COVID-19 vaccines (Pfizer-BioNTech and Moderna): update from the Advisory Committee on Immunization Practices—United States, July 2021. MMWR Morb Mortal Wkly Rep 70:1094–1099. doi:10.15585/mmwr.mm7032e4. 34383735 PMC8360272

[148] Pottegård A, Lund LC, Karlstad Ø, Dahl J, Andersen M, Hallas J, Lidegaard Ø, Tapia G, Gulseth HL, Ruiz PL-D, Watle SV, Mikkelsen AP, Pedersen L, Sørensen HT, Thomsen RW, Hviid A. 2021. Arterial events, venous thromboembolism, thrombocytopenia, and bleeding after vaccination with Oxford-AstraZeneca ChAdOx1-S in Denmark and Norway: population based cohort study. BMJ 373:n1114. doi:10.1136/bmj.n1114. 33952445 PMC8097496

[149] Chan B, Odutayo A, Juni P, Stall NM, Bobos P, Brown AD, Grill A, Ivers N, Maltsev A, McGeer A, Miller KJ, Niel U, Razak F, Sander B, Sholzberg M, Slutsky AS, Morris AM, Pai M, Ontario COVID-19 Science Advisory Table. 11 May 2021. Risk of vaccine-induced thrombotic thrombocytopenia (VITT) following the AstraZeneca/COVISHIELD adenovirus vector COVID-19 vaccines. https://covid19-sciencetable.ca/sciencebrief/risk-of-vaccine-induced-thrombotic-thrombocytopenia-vitt-following-the-astrazeneca-covishield-adenovirus-vector-covid-19-vaccines/.

[150] Baker AT, Boyd RJ, Sarkar D, Teijeira-Crespo A, Chan CK, Bates E, Waraich K, Vant J, Wilson E, Truong CD, Lipka-Lloyd M, Fromme P, Vermaas J, Williams D, Machiesky L, Heurich M, Nagalo BM, Coughlan L, Umlauf S, Chiu P-L, Rizkallah PJ, Cohen TS, Parker AL, Singharoy A, Borad MJ. 2021. ChAdOx1 interacts with CAR and PF4 with implications for thrombosis with thrombocytopenia syndrome. Sci Adv 7:eabl8213. doi:10.1126/sciadv.abl8213. 34851659 PMC8635433

[151] Schultz NH, Sørvoll IH, Michelsen AE, Munthe LA, Lund-Johansen F, Ahlen MT, Wiedmann M, Aamodt A-H, Skattør TH, Tjønnfjord GE, Holme PA. 2021. Thrombosis and thrombocytopenia after ChAdOx1 nCoV-19 vaccination. N Engl J Med 384:2124–2130. doi:10.1056/NEJMoa2104882. 33835768 PMC8112568

[152] EMA. 18 March 2021. COVID-19 vaccine AstraZeneca: benefits still outweigh the risks despite possible link to rare blood clots with low platelets. https://www.ema.europa.eu/en/news/covid-19-vaccine-astrazeneca-benefits-still-outweigh-risks-despite-possible-link-rare-blood-clots. Accessed 10 October 2022.

[153] Miesbach W, Makris M. 2020. COVID-19: coagulopathy, risk of thrombosis, and the rationale for anticoagulation. Clin Appl Thromb Hemost 26:107602962093814. doi:10.1177/1076029620938149. PMC7370334 32677459

[154] Verbeke R, Lentacker I, De Smedt SC, Dewitte H. 2019. Three decades of messenger RNA vaccine development. Nano Today 28:100766. doi:10.1016/j.nantod.2019.100766.

[155] Schlake T, Thess A, Fotin-Mleczek M, Kallen K-J. 2012. Developing mRNA-vaccine technologies. RNA Biol 9:1319–1330. doi:10.4161/rna.22269. 23064118 PMC3597572

[156] Martinon F, Krishnan S, Lenzen G, Magné R, Gomard E, Guillet J-G, Lévy J-P, Meulien P. 1993. Induction of virus-specific cytotoxic T lymphocytesin vivo by liposome-entrapped mRNA. Eur J Immunol 23:1719–1722. doi:10.1002/eji.1830230749. 8325342

[157] Zhang C, Maruggi G, Shan H, Li J. 2019. Advances in mRNA vaccines for infectious diseases. Front Immunol 10:594. doi:10.3389/fimmu.2019.00594. 30972078 PMC6446947

[158] Reichmuth AM, Oberli MA, Jaklenec A, Langer R, Blankschtein D. 2016. mRNA vaccine delivery using lipid nanoparticles. Ther Deliv 7:319–334. doi:10.4155/tde-2016-0006. 27075952 PMC5439223

[159] Iavarone C, O’hagan DT, Yu D, Delahaye NF, Ulmer JB. 2017. Mechanism of action of mRNA-based vaccines. Expert Rev Vaccines 16:871–881. doi:10.1080/14760584.2017.1355245. 28701102

[160] PHG Foundation. October 2018. RNA vaccines: an introduction. https://www.phgfoundation.org/briefing/rna-vaccines. Accessed 8 February 2021.

[161] Crotty S. 2014. T follicular helper cell differentiation, function, and roles in disease. Immunity 41:529–542. doi:10.1016/j.immuni.2014.10.004. 25367570 PMC4223692

[162] Pardi N, Hogan MJ, Porter FW, Weissman D. 2018. mRNA vaccines—a new era in vaccinology. Nat Rev Drug Discov 17:261–279. doi:10.1038/nrd.2017.243. 29326426 PMC5906799

[163] Stuart LM. 2021. In gratitude for mRNA vaccines. N Engl J Med 385:1436–1438. doi:10.1056/NEJMcibr2111445. 34569728

[164] Pardi N, Hogan MJ, Weissman D. 2020. Recent advances in mRNA vaccine technology. Curr Opin Immunol 65:14–20. doi:10.1016/j.coi.2020.01.008. 32244193

[165] Amanat F, Krammer F. 2020. SARS-CoV-2 vaccines: status report. Immunity 52:583–589. doi:10.1016/j.immuni.2020.03.007. 32259480 PMC7136867

[166] Fonteilles-Drabek S, Reddy D, Wells TNC. 2017. Managing intellectual property to develop medicines for the world’s poorest. Nat Rev Drug Discov 16:223–224. doi:10.1038/nrd.2017.24. 28232725

[167] GlaxoSmithKline. 2012. Evaluation of the kinetics of mRNA expression after two doses of GSK Biologicals’ candidate tuberculosis (tuberculosis) vaccine GSK 692342 in healthy adults. NCT01669096. https://clinicaltrials.gov/ct2/show/NCT01669096. Accessed 8 February 2021.

[168] Pardi N, Parkhouse K, Kirkpatrick E, McMahon M, Zost SJ, Mui BL, Tam YK, Karikó K, Barbosa CJ, Madden TD, Hope MJ, Krammer F, Hensley SE, Weissman D. 2018. Nucleoside-modified mRNA immunization elicits influenza virus hemagglutinin stalk-specific antibodies. Nat Commun 9:3361. doi:10.1038/s41467-018-05482-0. 30135514 PMC6105651

[169] Veiga N, Goldsmith M, Granot Y, Rosenblum D, Dammes N, Kedmi R, Ramishetti S, Peer D. 2018. Cell specific delivery of modified mRNA expressing therapeutic proteins to leukocytes. Nat Commun 9:4493. doi:10.1038/s41467-018-06936-1. 30374059 PMC6206083

[170] Vabret N, Britton GJ, Gruber C, Hegde S, Kim J, Kuksin M, Levantovsky R, Malle L, Moreira A, Park MD, Pia L, Risson E, Saffern M, Salomé B, Esai Selvan M, Spindler MP, Tan J, van der Heide V, Gregory JK, Alexandropoulos K, Bhardwaj N, Brown BD, Greenbaum B, Gümüş ZH, Homann D, Horowitz A, Kamphorst AO, Curotto de Lafaille MA, Mehandru S, Merad M, Samstein RM, Sinai Immunology Review Project. 2020. Immunology of COVID-19: current state of the science. Immunity 52:910–941. doi:10.1016/j.immuni.2020.05.002. 32505227 PMC7200337

[171] Chien KR, Zangi L, Lui KO. 2014. Synthetic chemically modified mRNA (modRNA): toward a new technology platform for cardiovascular biology and medicine. Cold Spring Harb Perspect Med 5:a014035. doi:10.1101/cshperspect.a014035. 25301935 PMC4292072

[172] Pfizer. 2020. Pfizer and BioNTech announce early positive data from an ongoing phase 1/2 study of mRNA-based vaccine candidate against SARS-CoV-2. https://www.pfizer.com/news/press-release/press-release-detail/pfizer-and-biontech-announce-early-positive-data-ongoing-0. Accessed 8 February 2021.

[173] National Institute of Allergy and Infectious Diseases. 2020. Phase I, open-label, dose-ranging study of the safety and immunogenicity of 2019-nCoV vaccine (mRNA-1273) in healthy adults. NCT04283461. https://clinicaltrials.gov/ct2/show/NCT04283461.

[174] Funk CD, Laferrière C, Ardakani A. 2020. A snapshot of the global race for vaccines targeting SARS-CoV-2 and the COVID-19 pandemic. Front Pharmacol 11:937. doi:10.3389/fphar.2020.00937. 32636754 PMC7317023

[175] Ledford H. 2021. What the Moderna–NIH COVID vaccine patent fight means for research. Nature 600:200–201. doi:10.1038/d41586-021-03535-x. 34853416

[176] Diamond D. 17 December 2021. Moderna halts patent fight over coronavirus vaccine with federal government. Washington Post, Washington, DC. https://www.washingtonpost.com/health/2021/12/17/moderna-vaccine-patent-dispute-nih/.

[177] Sealy A, Goodman B. 26 August 2022. Moderna files patent infringement lawsuits against Pfizer and BioNTech over mRNA Covid-19 vaccines. CNN. https://www.cnn.com/2022/08/26/health/moderna-pfizer-mrna-patent-lawsuit/index.html. Accessed 10 October 2022.

[178] Polack FP, Thomas SJ, Kitchin N, Absalon J, Gurtman A, Lockhart S, Perez JL, Pérez Marc G, Moreira ED, Zerbini C, Bailey R, Swanson KA, Roychoudhury S, Koury K, Li P, Kalina WV, Cooper D, Frenck RW, Hammitt LL, Türeci Ö, Nell H, Schaefer A, Ünal S, Tresnan DB, Mather S, Dormitzer PR, Şahin U, Jansen KU, Gruber WC, C4591001 Clinical Trial Group. 2020. Safety and Efficacy of the BNT162b2 mRNA Covid-19 Vaccine. N Engl J Med 383:2603–2615. doi:10.1056/NEJMoa2034577. 33301246 PMC7745181

[179] Baden LR, El Sahly HM, Essink B, Kotloff K, Frey S, Novak R, Diemert D, Spector SA, Rouphael N, Creech CB, McGettigan J, Khetan S, Segall N, Solis J, Brosz A, Fierro C, Schwartz H, Neuzil K, Corey L, Gilbert P, Janes H, Follmann D, Marovich M, Mascola J, Polakowski L, Ledgerwood J, Graham BS, Bennett H, Pajon R, Knightly C, Leav B, Deng W, Zhou H, Han S, Ivarsson M, Miller J, Zaks T. 2021. Efficacy and safety of the mRNA-1273 SARS-CoV-2 vaccine. N Engl J Med 384:403–416. doi:10.1056/NEJMoa2035389. 33378609 PMC7787219

[180] FDA. 11 December 2020. FDA takes key action in fight against COVID-19 by issuing emergency use authorization for first COVID-19 vaccine. FDA, Silver Spring, MD. https://www.fda.gov/news-events/press-announcements/fda-takes-key-action-fight-against-covid-19-issuing-emergency-use-authorization-first-covid-19. Accessed 8 February 2021.

[181] Oliver SE, Gargano JW, Marin M, Wallace M, Curran KG, Chamberland M, McClung N, Campos-Outcalt D, Morgan RL, Mbaeyi S, Romero JR, Talbot HK, Lee GM, Bell BP, Dooling K. 2021. The Advisory Committee on Immunization Practices’ interim recommendation for use of Moderna COVID-19 vaccine—United States, December 2020. MMWR Morbid Mortal Wkly Rep 69:1653–1656. doi:10.15585/mmwr.mm695152e1. PMC9191904 33382675

[182] Zimmer C, Corum J, Wee S-L, Kristoffersen M. 2020. Coronavirus vaccine tracker. The New York Times, New York, NY. https://www.nytimes.com/interactive/2020/science/coronavirus-vaccine-tracker.html.

[183] Thompson MG, Burgess JL, Naleway AL, Tyner H, Yoon SK, Meece J, Olsho LEW, Caban-Martinez AJ, Fowlkes AL, Lutrick K, Groom HC, Dunnigan K, Odean MJ, Hegmann K, Stefanski E, Edwards LJ, Schaefer-Solle N, Grant L, Ellingson K, Kuntz JL, Zunie T, Thiese MS, Ivacic L, Wesley MG, Mayo Lamberte J, Sun X, Smith ME, Phillips AL, Groover KD, Yoo YM, Gerald J, Brown RT, Herring MK, Joseph G, Beitel S, Morrill TC, Mak J, Rivers P, Poe BP, Lynch B, Zhou Y, Zhang J, Kelleher A, Li Y, Dickerson M, Hanson E, Guenther K, Tong S, Bateman A, Reisdorf E, et al. 2021. Prevention and attenuation of Covid-19 with the BNT162b2 and mRNA-1273 vaccines. N Engl J Med 385:320–329. doi:10.1056/NEJMoa2107058. 34192428 PMC8262622

[184] World Health Organization. 1 March 2021. The effects of virus variants on COVID-19 vaccines. https://www.who.int/news-room/feature-stories/detail/the-effects-of-virus-variants-on-covid-19-vaccines. Accessed 10 October 2022.

[185] Puranik A, Lenehan PJ, Silvert E, Niesen MJM, Corchado-Garcia J, O’Horo JC, Virk A, Swift MD, Halamka J, Badley AD, Venkatakrishnan AJ, Soundararajan V. 2021. Comparison of two highly-effective mRNA vaccines for COVID-19 during periods of Alpha and Delta variant prevalence. medRxiv. doi:10.1101/2021.08.06.21261707.

[186] Tseng HF, Ackerson BK, Luo Y, Sy LS, Talarico CA, Tian Y, Bruxvoort KJ, Tubert JE, Florea A, Ku JH, Lee GS, Choi SK, Takhar HS, Aragones M, Qian L. 2022. Effectiveness of mRNA-1273 against SARS-CoV-2 Omicron and Delta variants. Nat Med 28:1063–1071. doi:10.1038/s41591-022-01753-y. 35189624 PMC9117141

[187] Collie S, Champion J, Moultrie H, Bekker L-G, Gray G. 2022. Effectiveness of BNT162b2 vaccine against Omicron variant in South Africa. N Engl J Med 386:494–496. doi:10.1056/NEJMc2119270. 34965358 PMC8757569

[188] Andrews N, Stowe J, Kirsebom F, Toffa S, Rickeard T, Gallagher E, Gower C, Kall M, Groves N, O’Connell A-M, Simons D, Blomquist PB, Zaidi A, Nash S, Iwani Binti Abdul Aziz N, Thelwall S, Dabrera G, Myers R, Amirthalingam G, Gharbia S, Barrett JC, Elson R, Ladhani SN, Ferguson N, Zambon M, Campbell CNJ, Brown K, Hopkins S, Chand M, Ramsay M, Lopez Bernal J. 2022. Covid-19 vaccine effectiveness against the Omicron (B.1.1.529) variant. N Engl J Med 386:1532–1546. doi:10.1056/NEJMoa2119451. 35249272 PMC8908811

[189] The New York Times. 2021. Coronavirus in the U.S.: latest map and case count. The New York Times, New York, NY. https://www.nytimes.com/interactive/2021/us/covid-cases.html. Accessed 11 March 2022.

[190] Tan S. 7 January 2022. Four charts that analyze how omicron’s wave compares to previous coronavirus peaks. Washington Post, Washington, DC. https://www.washingtonpost.com/health/interactive/2022/omicron-comparison-cases-deaths-hospitalizations/. Accessed 10 October 2022.

[191] Abu Mouch S, Roguin A, Hellou E, Ishai A, Shoshan U, Mahamid L, Zoabi M, Aisman M, Goldschmid N, Berar Yanay N. 2021. Myocarditis following COVID-19 mRNA vaccination. Vaccine 39:3790–3793. doi:10.1016/j.vaccine.2021.05.087. 34092429 PMC8162819

[192] Kim HW, Jenista ER, Wendell DC, Azevedo CF, Campbell MJ, Darty SN, Parker MA, Kim RJ. 2021. Patients with acute myocarditis following mRNA COVID-19 vaccination. JAMA Cardiol 6:1196–1201. doi:10.1001/jamacardio.2021.2828. 34185046 PMC8243258

[193] Mevorach D, Anis E, Cedar N, Bromberg M, Haas EJ, Nadir E, Olsha-Castell S, Arad D, Hasin T, Levi N, Asleh R, Amir O, Meir K, Cohen D, Dichtiar R, Novick D, Hershkovitz Y, Dagan R, Leitersdorf I, Ben-Ami R, Miskin I, Saliba W, Muhsen K, Levi Y, Green MS, Keinan-Boker L, Alroy-Preis S. 2021. Myocarditis after BNT162b2 mRNA vaccine against Covid-19 in Israel. N Engl J Med 385:2140–2149. doi:10.1056/NEJMoa2109730. 34614328 PMC8531987

[194] Goddard K, Hanson KE, Lewis N, Weintraub E, Fireman B, Klein NP. 2022. Incidence of myocarditis/pericarditis following mRNA COVID-19 vaccination among children and younger adults in the United States. Ann Intern Med 175:1169–1771. doi:10.7326/m22-2274. 36191323 PMC9578536

[195] Matta A, Kunadharaju R, Osman M, Jesme C, McMiller Z, Johnson EM, Matta D, Kallamadi R, Bande D. 2021. Clinical presentation and outcomes of myocarditis post mRNA vaccination: a meta-analysis and systematic review. Cureus 13:e19240. doi:10.7759/cureus.19240. 34877217 PMC8641964

[196] Kim JH, Marks F, Clemens JD. 2021. Looking beyond COVID-19 vaccine phase 3 trials. Nat Med 27:205–211. doi:10.1038/s41591-021-01230-y. 33469205

[197] FDA. 30 June 2022. Coronavirus (COVID-19) update: FDA recommends inclusion of Omicron BA.4/5 component for COVID-19 vaccine booster doses. FDA, Silver Spring, MD. https://www.fda.gov/news-events/press-announcements/coronavirus-covid-19-update-fda-recommends-inclusion-omicron-ba45-component-covid-19-vaccine-booster. Accessed 10 October 2022.

[198] Frieden J. 29 June 2022. FDA panel gives thumbs up to Omicron-containing COVID boosters. https://www.medpagetoday.com/infectiousdisease/covid19vaccine/99493. Accessed 10 October 2022.

[199] FDA. 30 June 2022. Fall 2022 COVID-19 vaccine strain composition selection recommendation. FDA, Silver Spring, MD. https://www.fda.gov/media/159597/download.

[200] Erman M. 30 June 2022. U.S. FDA to use existing Omicron booster data to review shots targeting new subvariants—official. Reuters, London, United Kingdom. https://www.reuters.com/business/healthcare-pharmaceuticals/fda-will-not-require-clinical-trial-data-authorize-redesigned-covid-boosters-2022-06-30/.

[201] Tseng HF, Ackerson BK, Bruxvoort KJ, Sy LS, Tubert JE, Lee GS, Ku JH, Florea A, Luo Y, Qiu S, Choi SK, Takhar HS, Aragones M, Paila YD, Chavers S, Qian L. 1 October 2022. Effectiveness of mRNA-1273 against infection and COVID-19 hospitalization with SARS-CoV-2 Omicron subvariants: BA.1, BA.2, BA.2.12.1, BA.4, and BA.5. medRxiv. doi:10.1101/2022.09.30.22280573.

[202] Hardt K, Vandebosch A, Sadoff J, Gars ML, Truyers C, Lowson D, Van Dromme I, Vingerhoets J, Kamphuis T, Scheper G, Ruiz-Guiñazú J, Faust SN, Spinner CD, Schuitemaker H, Van Hoof J, Douoguih M, Struyf F. 1 January 2022. Efficacy and safety of a booster regimen of Ad26.COV2.S vaccine against Covid-19. medRxiv. doi:10.1101/2022.01.28.22270043.

[203] Dolzhikova I, Iliukhina A, Kovyrshina A, Kuzina A, Gushchin V, Siniavin A, Pochtovyi A, Shidlovskaya E, Kuznetsova N, Megeryan M, Dzharullaeva A, Erokhova A, Izhaeva F, Grousova D, Botikov A, Shcheblyakov D, Tukhvatulin A, Zubkova O, Logunov D, Gintsburg A. 21 December 2021. Sputnik Light booster after Sputnik V vaccination induces robust neutralizing antibody response to B.1.1.529 (Omicron) SARS-CoV-2 variant. medRxiv. doi:10.1101/2021.12.17.21267976.

[204] Flaxman A, Marchevsky NG, Jenkin D, Aboagye J, Aley PK, Angus B, Belij-Rammerstorfer S, Bibi S, Bittaye M, Cappuccini F, Cicconi P, Clutterbuck EA, Davies S, Dejnirattisai W, Dold C, Ewer KJ, Folegatti PM, Fowler J, Hill AVS, Kerridge S, Minassian AM, Mongkolsapaya J, Mujadidi YF, Plested E, Ramasamy MN, Robinson H, Sanders H, Sheehan E, Smith H, Snape MD, Song R, Woods D, Screaton G, Gilbert SC, Voysey M, Pollard AJ, Lambe T, Adlou S, Aley R, Ali A, Anslow R, Baker M, Baker P, Barrett JR, Bates L, Beadon K, Beckley R, Bell J, Bellamy D, Beveridge A, Oxford COVID Vaccine Trial group, et al. 2021. Reactogenicity and immunogenicity after a late second dose or a third dose of ChAdOx1 nCoV-19 in the UK: a substudy of two randomised controlled trials (COV001 and COV002). Lancet 398:981–990. doi:10.1016/S0140-6736(21)01699-8. 34480858 PMC8409975

[205] Regev-Yochay G, Gonen T, Gilboa M, Mandelboim M, Indenbaum V, Amit S, Meltzer L, Asraf K, Cohen C, Fluss R, Biber A, Nemet I, Kliker L, Joseph G, Doolman R, Mendelson E, Freedman LS, Harats D, Kreiss Y, Lustig Y. 15 February 2022. 4th dose COVID mRNA vaccines’ immunogenicity & efficacy against Omicron VOC. medRxiv. doi:10.1101/2022.02.15.22270948.

[206] Chiu N-C, Chi H, Tu Y-K, Huang Y-N, Tai Y-L, Weng S-L, Chang L, Huang DT-N, Huang F-Y, Lin C-Y. 2021. To mix or not to mix? A rapid systematic review of heterologous prime–boost covid-19 vaccination. Expert Rev Vaccines 20:1211–1220. doi:10.1080/14760584.2021.1971522. 34415818 PMC8425437

[207] Atmar RL, Lyke KE, Deming ME, Jackson LA, Branche AR, El Sahly HM, Rostad CA, Martin JM, Johnston C, Rupp RE, Mulligan MJ, Brady RC, Frenck RW, Bäcker M, Kottkamp AC, Babu TM, Rajakumar K, Edupuganti S, Dobrzynski D, Coler RN, Posavad CM, Archer JI, Crandon S, Nayak SU, Szydlo D, Zemanek JA, Dominguez Islas CP, Brown ER, Suthar MS, McElrath MJ, McDermott AB, O’Connell SE, Montefiori DC, Eaton A, Neuzil KM, Stephens DS, Roberts PC, Beigel JH, DMID 21–0012 Study Group. 2022. Homologous and heterologous Covid-19 booster vaccinations. N Engl J Med 386:1046–1057. doi:10.1056/NEJMoa2116414. 35081293 PMC8820244

[208] Jara A, Undurraga EA, Zubizarreta JR, González C, Pizarro A, Acevedo J, Leo K, Paredes F, Bralic T, Vergara V, Mosso M, Leon F, Parot I, Leighton P, Suárez P, Rios JC, García-Escorza H, Araos R. 2022. Effectiveness of homologous and heterologous booster doses for an inactivated SARS-CoV-2 vaccine: a large-scale prospective cohort study. Lancet Glob Health 10:e798–e806. doi:10.1016/S2214-109X(22)00112-7. 35472300 PMC9034854

[209] Sapkota B, Saud B, Shrestha R, Al-Fahad D, Sah R, Shrestha S, Rodriguez-Morales AJ. 2022. Heterologous prime–boost strategies for COVID-19 vaccines. J Travel Med 29:taab191. doi:10.1093/jtm/taab191. 34918097 PMC8754745

[210] Assawakosri S, Kanokudom S, Suntronwong N, Auphimai C, Nilyanimit P, Vichaiwattana P, Thongmee T, Duangchinda T, Chantima W, Pakchotanon P, Srimuan D, Thatsanatorn T, Klinfueng S, Yorsaeng R, Sudhinaraset N, Wanlapakorn N, Mongkolsapaya J, Honsawek S, Poovorawan Y. 2022. Neutralizing activities against the omicron variant after a heterologous booster in healthy adults receiving two doses of CoronaVac vaccination. J Infect Dis 226:1372–1381. doi:10.1093/infdis/jiac092. 35267040

[211] Accorsi EK, Britton A, Shang N, Fleming-Dutra KE, Link-Gelles R, Smith ZR, Derado G, Miller J, Schrag SJ, Verani JR. 2022. Effectiveness of homologous and heterologous Covid-19 boosters against Omicron. N Engl J Med 386:2433–2435. doi:10.1056/NEJMc2203165. 35613039 PMC9165559

[212] Munro APS, Janani L, Cornelius V, Aley PK, Babbage G, Baxter D, Bula M, Cathie K, Chatterjee K, Dodd K, Enever Y, Gokani K, Goodman AL, Green CA, Harndahl L, Haughney J, Hicks A, van der Klaauw AA, Kwok J, Lambe T, Libri V, Llewelyn MJ, McGregor AC, Minassian AM, Moore P, Mughal M, Mujadidi YF, Murira J, Osanlou O, Osanlou R, Owens DR, Pacurar M, Palfreeman A, Pan D, Rampling T, Regan K, Saich S, Salkeld J, Saralaya D, Sharma S, Sheridan R, Sturdy A, Thomson EC, Todd S, Twelves C, Read RC, Charlton S, Hallis B, Ramsay M, Andrews N, et al. 2021. Safety and immunogenicity of seven COVID-19 vaccines as a third dose (booster) following two doses of ChAdOx1 nCov-19 or BNT162b2 in the UK (COV-BOOST): a blinded, multicentre, randomised, controlled, phase 2 trial. Lancet 398:2258–2276. doi:10.1016/S0140-6736(21)02717-3. 34863358 PMC8639161

[213] European Centre for Disease Prevention and Control. 18 May 2021. Overview of EU/EEA country recommendations on COVID-19 vaccination with Vaxzevria, and a scoping review of evidence to guide decision-making. European Centre for Disease Prevention and Control, Solna, Sweden. https://www.ecdc.europa.eu/en/publications-data/overview-eueea-country-recommendations-covid-19-vaccination-vaxzevria-and-scoping. Accessed 10 October 2022.

[214] Duarte-Salles T, Prieto-Alhambra D. 2021. Heterologous vaccine regimens against COVID-19. Lancet 398:94–95. doi:10.1016/S0140-6736(21)01442-2. 34181881 PMC8233006

[215] Moderna. 8 June 2022. Moderna announces Omicron-containing bivalent booster candidate mRNA-1273.214 demonstrates superior antibody response against Omicron. Moderna, Cambridge, MA. https://investors.modernatx.com/news/news-details/2022/Moderna-Announces-Omicron-Containing-Bivalent-Booster-Candidate-mRNA-1273.214-Demonstrates-Superior-Antibody-Response-Against-Omicron/default.aspx. Accessed 10 October 2022.

[216] Choi A, Koch M, Wu K, Chu L, Ma L, Hill A, Nunna N, Huang W, Oestreicher J, Colpitts T, Bennett H, Legault H, Paila Y, Nestorova B, Ding B, Montefiori D, Pajon R, Miller JM, Leav B, Carfi A, McPhee R, Edwards DK. 2021. Safety and immunogenicity of SARS-CoV-2 variant mRNA vaccine boosters in healthy adults: an interim analysis. Nat Med 27:2025–2031. doi:10.1038/s41591-021-01527-y. 34526698 PMC8604720

[217] Pajon R, Doria-Rose NA, Shen X, Schmidt SD, O’Dell S, McDanal C, Feng W, Tong J, Eaton A, Maglinao M, Tang H, Manning KE, Edara V-V, Lai L, Ellis M, Moore KM, Floyd K, Foster SL, Posavad CM, Atmar RL, Lyke KE, Zhou T, Wang L, Zhang Y, Gaudinski MR, Black WP, Gordon I, Guech M, Ledgerwood JE, Misasi JN, Widge A, Sullivan NJ, Roberts PC, Beigel JH, Korber B, Baden LR, El Sahly H, Chalkias S, Zhou H, Feng J, Girard B, Das R, Aunins A, Edwards DK, Suthar MS, Mascola JR, Montefiori DC. 2022. SARS-CoV-2 Omicron variant neutralization after mRNA-1273 booster vaccination. N Engl J Med 386:1088–1091. doi:10.1056/NEJMc2119912. 35081298 PMC8809504

[218] Pfizer. 25 June 2022. Pfizer and BioNTech announce Omicron-adapted COVID-19 vaccine candidates demonstrate high immune response against Omicron. Pfizer, New York, NY. https://www.pfizer.com/news/press-release/press-release-detail/pfizer-and-biontech-announce-omicron-adapted-covid-19. Accessed 10 October 2022.

[219] Moxon R, Reche PA, Rappuoli R. 2019. Editorial: Reverse vaccinology. Front Immunol 10:2776. doi:10.3389/fimmu.2019.02776. 31849959 PMC6901788

[220] Kudchodkar SB, Choi H, Reuschel EL, Esquivel R, Jin-Ah Kwon J, Jeong M, Maslow JN, Reed CC, White S, Kim JJ, Kobinger GP, Tebas P, Weiner DB, Muthumani K. 2018. Rapid response to an emerging infectious disease—lessons learned from development of a synthetic DNA vaccine targeting Zika virus. Microbes Infect 20:676–684. doi:10.1016/j.micinf.2018.03.001. 29555345 PMC6593156

[221] Olena A. 21 February 2020. Newer vaccine technologies deployed to develop COVID-19 shot. The Scientist Magazine, Wilmington, DE. https://www.the-scientist.com/news-opinion/newer-vaccine-technologies-deployed-to-develop-covid-19-shot-67152.

[222] Pulliam JRC, van Schalkwyk C, Govender N, von Gottberg A, Cohen C, Groome MJ, Dushoff J, Mlisana K, Moultrie H. 2 December 2021. Increased risk of SARS-CoV-2 reinfection associated with emergence of Omicron in South Africa. medRxiv. doi:10.1101/2021.11.11.21266068. PMC8995029 35289632

[223] Centers for Disease Control and Prevention. 2 December 2021. Science brief: Omicron (B.1.1.529) variant. Centers for Disease Control and Prevention, Atlanta, GA. https://public4.pagefreezer.com/browse/CDC%20Covid%20Pages/15-07-2022T12:20/https://www.cdc.gov/coronavirus/2019-ncov/science/science-briefs/scientific-brief-omicron-variant.html. Accessed 10 October 2022.

[224] Planas D, Bruel T, Grzelak L, Guivel-Benhassine F, Staropoli I, Porrot F, Planchais C, Buchrieser J, Rajah MM, Bishop E, Albert M, Donati F, Prot M, Behillil S, Enouf V, Maquart M, Smati-Lafarge M, Varon E, Schortgen F, Yahyaoui L, Gonzalez M, De Sèze J, Péré H, Veyer D, Sève A, Simon-Lorière E, Fafi-Kremer S, Stefic K, Mouquet H, Hocqueloux L, van der Werf S, Prazuck T, Schwartz O. 2021. Sensitivity of infectious SARS-CoV-2 B.1.1.7 and B.1.351 variants to neutralizing antibodies. Nat Med 27:917–924. doi:10.1038/s41591-021-01318-5. 33772244

[225] Wang P, Nair MS, Liu L, Iketani S, Luo Y, Guo Y, Wang M, Yu J, Zhang B, Kwong PD, Graham BS, Mascola JR, Chang JY, Yin MT, Sobieszczyk M, Kyratsous CA, Shapiro L, Sheng Z, Huang Y, Ho DD. 2021. Antibody resistance of SARS-CoV-2 variants B.1.351 and B.1.1.7. Nature 593:130–135. doi:10.1038/s41586-021-03398-2. 33684923

[226] Edara VV, Hudson WH, Xie X, Ahmed R, Suthar MS. 2021. Neutralizing antibodies against SARS-CoV-2 variants after infection and vaccination. JAMA 325:1896–1898. doi:10.1001/jama.2021.4388. 33739374 PMC7980146

[227] World Health Organization. 26 November 2021. Classification of Omicron (B.1.1.529): SARS-CoV-2 variant of concern. https://www.who.int/news/item/26-11-2021-classification-of-omicron-(b.1.1.529)-sars-cov-2-variant-of-concern. Accessed 10 October 2022.

[228] Centers for Disease Control and Prevention. 2020. COVID data tracker. Centers for Disease Control and Prevention, Atlanta, GA. https://covid.cdc.gov/covid-data-tracker. Accessed 10 October 2022.

[229] Kuhlmann C, Mayer CK, Claassen M, Maponga TG, Sutherland AD, Suliman T, Shaw M, Preiser W. 10 December 2021. Breakthrough infections with SARS-CoV-2 Omicron variant despite booster dose of mRNA vaccine. SSRN J doi:10.2139/ssrn.3981711. PMC8765759 35063123

[230] Self WH, Tenforde MW, Rhoads JP, Gaglani M, Ginde AA, Douin DJ, Olson SM, Talbot HK, Casey JD, Mohr NM, Zepeski A, McNeal T, Ghamande S, Gibbs KW, Files DC, Hager DN, Shehu A, Prekker ME, Erickson HL, Gong MN, Mohamed A, Henning DJ, Steingrub JS, Peltan ID, Brown SM, Martin ET, Monto AS, Khan A, Hough CL, Busse LW, ten Lohuis CC, Duggal A, Wilson JG, Gordon AJ, Qadir N, Chang SY, Mallow C, Rivas C, Babcock HM, Kwon JH, Exline MC, Halasa N, Chappell JD, Lauring AS, Grijalva CG, Rice TW, Jones ID, Stubblefield WB, Baughman A, Womack KN, et al. 2021. Comparative effectiveness of Moderna, Pfizer-BioNTech, and Janssen (Johnson & Johnson) vaccines in preventing COVID-19 hospitalizations among adults without immunocompromising conditions—United States, March–August 2021. MMWR Morb Mortal Wkly Rep 70:1337–1343. doi:10.15585/mmwr.mm7038e1. 34555004 PMC8459899

[231] Mueller B, Robbins R. 2 August 2021. Where a vast global vaccination program went wrong. The New York Times, New York, NY. https://www.nytimes.com/2021/08/02/world/europe/covax-covid-vaccine-problems-africa.html.

[232] Sheridan C. 2021. Innovators target vaccines for variants and shortages in global South. Nat Biotechnol 39:393–396. doi:10.1038/d41587-021-00001-x. 33731936

[233] Nohynek H, Wilder-Smith A. 2022. Does the world still need new Covid-19 vaccines? N Engl J Med 386:2140–2142. doi:10.1056/NEJMe2204695. 35507476 PMC9093715

[234] BioRender. 2023. Human coronavirus structure. https://app.biorender.com/biorender-templates/figures/all/t-5f21e90283765600b08fbe9d-human-coronavirus-structure. Accessed 10 October 2022.

[235] National Institute of Allergy and Infectious Diseases. 13 February 2020. New images of novel coronavirus SARS-CoV-2 now available. National Institute of Allergy and Infectious Diseases, Bethesda, MD. https://www.niaid.nih.gov/news-events/novel-coronavirus-sarscov2-images. Retrieved 10 October 2022.

[236] Jordahl K, Van Den Bossche J, Fleischmann M, McBride J, Wasserman J, Badaracco AG, Gerard J, Snow AD, Tratner J, Perry M, Farmer C, Hjelle GA, Cochran M, Gillies S, Culbertson L, Bartos M, Ward B, Caria G, Taves M, Eubank N, Sangarshanan, Flavin J, Richards M, Rey S, Maxalbert, Bilogur A, Ren C, Arribas-Bel D, Mesejo-León D, Wasser L. 16 October 2021. geopandas/geopandas: v0.10.2. Zenodo. https://doi.org/gqkzpv.

[237] VIPER Group COVID19 Vaccine Tracker Team. 2022. COVID19 Vaccine Tracker. Types of vaccines. https://covid19.trackvaccines.org/types-of-vaccines/. Accessed 10 October 2022.

